# Interplay between malic enzyme 2, de novo serine synthesis, and the malate-aspartate shuttle drives metabolic adaptation in triple-negative breast cancer

**DOI:** 10.1186/s40170-025-00410-5

**Published:** 2025-10-14

**Authors:** Jin Heon Jeon, Mark D. Slayton, Ben Krinkel, Olamide Animasahun, Ajay Shankaran, Fulei Wuchu, Minal Nenwani, Zackariah Farah, Julia Burke, Abhinav Achreja, Brisilda Nilaj, Kerslee Kohagen, Yi-Hsien Eu, Alyssa Rosenfeld, Mason Collard, Liwei Bao, Xu Cheng, Celina Kleer, Christopher Squire, Kerry Loomes, Deepak Nagrath, Sofia D. Merajver

**Affiliations:** 1 Department of Internal Medicine, Michigan Medicine, University of Michigan, Ann Arbor, MI 48109 USA; 2 Rogel Cancer Center, Michigan Medicine, University of Michigan, MI 48109 Ann Arbor, United States; 3 Department of Biomedical Engineering, Michigan Medicine, University of Michigan, Ann Arbor, MI 48109 USA; 4 Department of Chemical Engineering, University of Michigan, MI 48109 Ann Arbor, United States; 5 Laboratory for Systems Biology of Human Diseases, University of Michigan, MI 48109 Ann Arbor, United States; 6 Biointerfaces Institute, University of Michigan, MI 48109 Ann Arbor, United States; 7 School of Biological Sciences, University of Auckland, Auckland, New Zealand

## Abstract

Triple-negative breast cancer (TNBC) is an aggressive and heterogeneous subtype of breast cancer with poor clinical outcomes. Malic enzyme 2 (ME2) is a mitochondrial enzyme that catalyzes the conversion of malate to pyruvate and has been proposed as a therapeutic target. ME2 is highly expressed in many cell types including TNBC cells. We sought to define the molecular and cellular consequences of ME2 inhibition to facilitate its clinical translation. Here, we systematically evaluated the cellular and molecular effects of ME2 knockdown (ME2kd) in multiple TNBC models. ME2kd had heterogeneous effects on proliferation, migration, and metabolic flexibility in TNBC cell lines. ME2kd MDA-MB-468 xenografts in nude mice grew significantly slower and conferred prolonged host survival. ME2kd caused distinct shifts in mitochondrial respiration and glycolysis, whereas metabolomic and transcriptomic analyses revealed altered tricarboxylic acid (TCA) cycle flux, glutamine consumption, and serine/glycine metabolism, partly through changes in malate-aspartate shuttle (MAS) activity. The interplay between ME2, the serine synthesis pathway and the MAS was investigated with metabolite deprivation and co-knockdown assays. Importantly, we determined the crystal structure of ME2 bound to the small-molecule inhibitor NPD-389 and identified the binding interactions that drive the inhibitory response. These findings help to clarify the role of ME2 in TNBC phenotypes and highlight the therapeutic potential of ME2 inhibition in precision oncology.

**Graphical Abstract:**

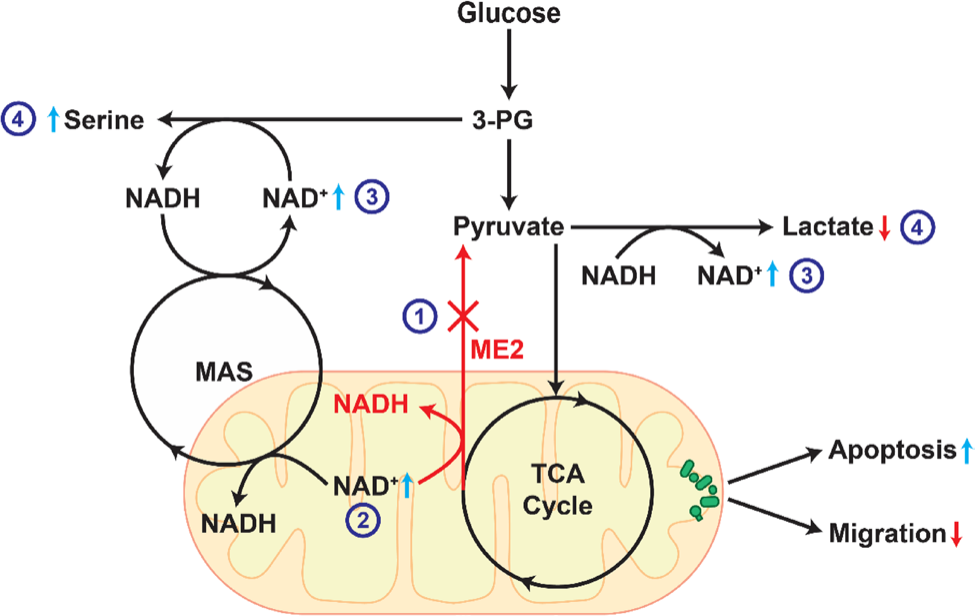

**Supplementary Information:**

The online version contains supplementary material available at 10.1186/s40170-025-00410-5.

## Introduction

Breast cancer is a prominent contributor to the global burden of cancer, causing 30% of cancers and being the second cause of deaths from cancer in women. Triple-negative breast cancer (TNBC), which lacks expression of ER/PR and does not overexpress HER2, is an aggressive, diverse subtype that comprises ~ 10–15% of cases, and is associated with poor prognosis due to high metastatic potential and limited treatments.

Metabolic reprogramming is a hallmark of cancer and alterations in cellular metabolism support rapid cell growth and proliferation. Shifts in metabolic pathway activities can render cancer cells vulnerable to metabolic enzyme inhibition, thus identifying potential therapeutic targets [1]. The inhibition of specific metabolic enzymes has shown promise in disrupting the metabolic plasticity that may be essential for cancer cell survival, especially when under stress from anti-cancer therapies [2, 3]. However, this approach remains challenging owing to the interpatient variability in tumor metabolite reliance.

Malic enzyme 2 (ME2) is one of three known isoforms of malic enzyme (ME1, ME2, and ME3), and one of two mitochondrial isoforms (ME2 and ME3). A major function of the malic enzymes is to convert malate to pyruvate, serving as a metabolic shunt to regulate TCA cycle activity under fluctuating external stresses. However, more recently, ME2 has gained more attention for its role in regulating energy metabolism and redox homeostasis via the conversion of NAD(P)^+^ to NAD(P)H [4, 5]. Furthermore, several papers have started to bring attention to ME2’s role in regulating the redox balance not only within the mitochondria, but also between the mitochondria and the cytosol through glycolysis [6–8]. Due to this flexible and comprehensive role, ME2 has increasingly become of interest in cancer as a potential therapeutic target [4].

The de novo serine synthesis pathway (SSP) is tightly linked to the interplay between mitochondrial and cytosolic redox and energy metabolism. We were particularly interested in this pathway due to its well-established role in many breast cancers, as well as its dependence on redox balance and its association with the mitochondria, both of which are characteristics closely linked to ME2 activity [9–11]. Serine is biosynthesized via the SSP, branching from glycolysis in the cytosol. The SSP contributes to one-carbon metabolism of the folate and methionine cycle across the mitochondria and cytosol to produce purines and glutathione for proliferation and redox regulation, respectively. For this reason, the production of serine is tightly regulated by phosphoglycerate dehydrogenase (PHGDH) which utilizes NAD^+^, and by TCA metabolites such as glutamate and α-ketoglutarate through phosphoserine aminotransferase 1 (PSAT1). The SSP is known to play a significant role in many breast cancers, with certain subsets having elevated levels of PHGDH expression [12, 13]. This has led SSP to be of high interest for therapeutic targeting [14, 15].

The mechanism by which SSP regulates NAD(P)H levels across the mitochondria is via the folate cycle, with the forward reaction increasing NADH and NADP^+^ levels in the mitochondria and cytosol, respectively [16, 17]. However, Broeks et al. recently discovered that the malate-aspartate shuttle (MAS) is important for SSP function through regulation of the NAD^+^/NADH ratio [18]. In this scenario, MAS effectively shuttles malate from the mitochondria to the cytosol by converting NAD^+^ to NADH in the mitochondria, and NADH to NAD^+^ in the cytosol. This leads to a redox-equivalent export of NAD^+^ to the cytosol and import of NADH into the mitochondria. Ultimately, the resulting increase in cytosolic NADH can lead to the inhibition of glycolytic flux and an increase in SSP flux [18, 19]. Regulation of energy metabolism and redox balance between the cytosol and mitochondrial by the MAS has been shown to increase drug resistance in certain cancer cells and has made it a target of therapeutic interest for many cancers [19, 20].

In this study, we show that ME2 plays a significant role in regulating glycolytic and SSP flux via the MAS in TNBC cell lines. We evaluate ME2 knockdown (ME2kd) in TNBC models, explore how ME2 inhibition disrupts cancer metabolism, and identify predictive response pathways. By investigating the direct and indirect effects of ME2 activity on cell growth and the rewiring of metabolic profiles, we sought to elucidate its role in TNBC and identify predictive markers for ME2kd as a therapeutic strategy. We characterized TNBC and control cell lines in vitro and in vivo for proliferation, migration, apoptotic activity, and metabolic transcriptome changes in response to ME2kd. We performed detailed metabolite analyses using mass spectrometry, showing that mitochondrial function is impaired, and present data supporting the alternative utilization of the SSP and MAS under ME2kd. We present a novel crystal structure of ME2 bound to the malic enzyme inhibitor NPD-389, revealing detailed enzyme-compound interactions, providing a structural rationale and platform for designing new ME2-targeting molecules.

## Materials and methods

### ME2 correlation with genes in Serine metabolic pathway

Transcriptomic analysis was performed on a subset of samples (only TNBC) from the Breast Invasive Carcinoma dataset generated by Ciriello et al. from cBioPortal [21–23], which was determined by a lack of estrogen and progesterone receptor expression and clinically negative HER2 expression. In other words, we selected only samples with negative entries for “ER status by IHC”, “PR status by IHC” and “HER2 FISH status” in the clinical data file. Next, we performed a Pearson correlation test between the malic enzyme paralogs (ME1, ME2, and ME3) and genes associated with serine and glycine metabolism. All analyses were performed using Python, version 3.11.4.

### Cell Lines, culture conditions and SiRNA transfection

TNBC cell lines (MDA-MB-468, RRID: CVCL_0419; Hs578T, RRID: CVCL_0332; HCC1806, RRID: CVCL_1258; HCC70, RRID: CVCL_1270; and BT-20, RRID: CVCL_0178) and a non-tumorigenic breast cell line (MCF10a, RRID: CVCL_0598) were used for comparative analysis. All cell lines used in this study were purchased from ATCC. Cell line identities were verified by STR profiling (ATCC) and routinely tested for mycoplasma contamination using MycoAlert Mycoplasma Detection Kit (Lonza). MDA-MB-468 and Hs578T were cultured in high glucose DMEM (Corning) supplemented with 10% fetal bovine serum (FBS), 1% antibiotic-antimycotic (Gibco), and 0.05% gentamicin. HCC1806 and HCC70 were cultured in RPMI-1640 (ATCC) with 10% FBS, 1% penicillin/streptomycin, and 0.05% gentamicin. BT-20 cells were cultured in EMEM with Earle’s salts (Corning), 10% FBS, 1% antibiotic-antimycotic, and 0.05% gentamicin. MCF10a cells were cultured in DMEM/F12 (Corning) with 5% horse serum, 1% penicillin/streptomycin, 1% amphotericin B, 0.05% gentamicin, 5 µg/ml insulin, 1 µg/ml hydrocortisone, 20ng/ml epidermal growth factor, and 100 µg/ml cholera toxin. All cell lines were cultured in a humidified atmosphere at 37 °C and 5% CO_2_. For experiments in the BioTek BioSpa 8 automated incubator, cells were maintained at 37 °C with 10% CO_2_. ME2-targeting and negative control siRNAs were purchased from Integrated DNA Technologies. Reverse transfection was performed using RNAiMAX (Thermo Fisher Scientific) in a 48-well plate at 50% confluence.

### Lentiviral transduction

Lentiviral transfer plasmids carrying either scrambled shRNA or ME2-targeting shRNA with co-expression of TurboRFP were purchased from Horizon Discovery. The lentivirus was prepared at the University of Michigan viral vector core using a third-generation propagation system. Transduction was performed in subconfluent cell cultures for 72 h. Expression of scrambled or ME2-targeting shRNA was induced by adding doxycycline (Sigma; 0.125 µg/ml) to the culture medium. Positive cells were identified by TurboRFP fluorescence; cell sorting to remove unmodified cells was performed using the NanoCellect WOLFG2 cell sorter.

### Confirmation of ME2 knockdown

For cells in culture and post-extraction tumors, knockdown efficiency was assessed by quantitative PCR (qPCR) with ME2-specific primers and western blot analysis. For qPCR, total RNA was extracted using DirectZol RNA Microprep (Zymo Research) and cDNA was synthesized using the High-Capacity cDNA Reverse Transcription Kit (Applied Biosystems). Transcript analysis was performed using the Applied Biosystems QuantStudio 3. ME2 protein levels were quantified by western blotting using antibodies specific to ME2 and beta-actin (Cell Signaling) and detected with an Azure Biosystems c300 imager.

### Growth rate Analysis, donut and caspase 3/7 assay

Cell proliferation was measured in a Biotek BioSpa 8 automated incubator using an Agilent Cytation 5 multi-mode reader. Cells were plated in 24-well plates and growth rates were monitored over a period of 72 h post-transfection of ME2 knockdown by siRNA. The cell number was quantified at each time point using the Cellpose 2.0 segmentation algorithm after training specific to each cell line (cellpose.org). Donut assays were performed in 96-well black-walled Nunc plates (Thermo Fisher Scientific) using Oris stoppers (Platypus Technologies). Cells were seeded to obtain 100% confluency after 24 h, at which point stoppers were removed, and migration was assessed over 72 h. Caspase-3/7 activity was measured using CellEvent Caspase-3/7 Green Detection Reagent (Thermo Fisher Scientific) to assess apoptosis following ME2 knockdown. Cells were seeded in 96-well plates at a density of 5 × 10⁴ cells per well in doxycycline-containing medium (125 ng/mL doxycycline). After 48 h, the CellEvent Caspase-3/7 reagent was added directly to the culture medium at a final concentration of 5 µM. After a 30-minute incubation at 37 °C, 5% CO₂, fluorescence was measured using a microplate reader and visualized via fluorescence microscopy, comparing scrambled to ME2kd. The fluorescence intensity was normalized to the total number of cells based on the TurboRFP signal. Background fluorescence was determined using cells that did not receive the reagents. All experiments were performed in triplicate, and statistical significance was assessed using a two-tailed Student’s t-test or ANOVA, where appropriate.

### Metabolomic transcriptome panels

The NanoString nCounter Metabolic Pathways Panel was used to analyze the expression of genes involved in various metabolic pathways in TNBC and control cell lines. For each sample, 100 ng of total RNA was hybridized with the nCounter^®^ Metabolic Pathways Panel CodeSet at 65 °C for 20 h. After hybridization, the samples were processed using the nCounter^®^ Prep Station, and data were collected using the nCounter^®^ Digital Analyzer. Transcript counts were analyzed using Rosalind (nanostring.rosalind.bio) and nSolver (NanoString).

### TNBC xenografts in nude mice

Female athymic nude mice, aged 6–8 weeks, were purchased from Charles River Laboratories. Mice were housed under pathogen-free conditions with a 12-hour light/dark cycle and provided with autoclaved food and doxycycline-containing water (400 µg/mL doxycycline, 2% sucrose) ad libitum. Mice were anesthetized with a solution of ketamine/xylazine (0.1 mg/0.01 mg per 1 g of body weight prior to the administration of cancer cells. For each mouse, 100 µL of the cell suspension was injected subcutaneously into the left flank; HCC70 suspensions contained 1 × 10^6^ cells and MDA-MB-468 suspensions contained 1.5 × 10^6^ cells. Four groups of 10 mice were established, with each group receiving either a cell line expressing scrambled shRNA or ME2-targeting shRNA. All animal procedures were approved by the University of Michigan Institutional Animal Care and Use Committee (IACUC) and were performed in accordance with institutional guidelines.

### Gas Chromatography-Mass spectrometry and metabolite quantification

Both scrambled and ME2 knockdown cell lines were treated with 125 ng/mL doxycycline in their respective media for 48 h. Following treatment, cells were washed three times with PBS and then cultured in either complete RPMI-1640 medium (supplemented with 10% FBS, 100 U/mL Pen-Strep, and 125 ng/mL doxycycline) or serine- and glycine-deprived RPMI-1640 medium containing 11.11 mM U-^13^C_6_-labeled D-glucose for 24 h (supplemented with 10% dialyzed FBS, 100 U/mL Pen-Strep, and 125 ng/mL doxycycline).

Metabolites were harvested by adding a mixture of methanol and distilled water (1:1, v/v) directly to the cells. The samples were phase-separated by adding an equal volume of chloroform. The polar (upper) aqueous layer was isolated and dried overnight at 4 °C using a SpeedVac concentrator. Dried metabolites were first derivatized with 2% methoxamine (MOX) in pyridine at 45 °C for 1 h, followed by a second derivatization step with N-methyl-N-(t-butyldimethylsilyl)-trifluoroacetamide (MTBSTFA) containing 1% t-butyldimethylchlorosilane (t-BDMCS) at 45 °C for 30 min.

Gas chromatography–mass spectrometry (GC-MS) analysis was conducted using helium as the carrier gas flowing at 1 mL/min. The injection volume ranged from 1 to 2 µL, with the inlet temperature set at 270 °C. The oven temperature was held at 100 °C for 11 min, then ramped at 3.5 °C/min to 255 °C, followed by an increase to 320 °C at 15 °C/min and held for an additional 3 min. A solvent delay of 6–10 min was used. The MS ion source and quadrupole temperatures were set at 230 °C and 150 °C, respectively. The mass spectrometer was operated in the scan mode (70–600 m/z). Metabolite concentrations were quantified based on standard curves generated concurrently with the experimental samples. All measured metabolite concentrations were normalized to the protein content of cells cultured under identical conditions, as determined by BCA protein assay. Isotopic enrichment was corrected for natural isotope abundance using IsoCorrectoR before further analysis. Labeling was quantified both as mean enrichment and as mass isotopologue distributions (MIDs). Mean enrichment is reported in the main figures; complete MIDs are presented in Supplementary Figures S5 and S6.

Additionally, intracellular concentrations of critical metabolites, including malate, glutamate, NAD^+^, NADH, NADP^+^, and NADPH, were quantified using Metabolite-Glo assay kits (Promega), according to the manufacturer’s instructions.

### Seahorse assay

Cellular metabolic activity was measured using an XFe96 extracellular flux analyzer (Agilent, Santa Clara, CA, USA). Both scrambled control and ME2 knockdown cell lines were treated with 125 ng/mL doxycycline for 48 h prior to seeding. The cells were then seeded into XFe96 cell culture plates at optimized densities: MDA-MB-468, 40,000 cells/well; BT-20, 20,000 cells/well, HCC70, 30,000 cells/well; Hs578T, 20,000 cells/well; HCC1806, 20,000 cells/well; and MCF10a, 20,000 cells/well. The cells were cultured in media containing 125 ng/mL doxycycline and incubated at 37 °C with 5% CO₂ for an additional 24 h to allow proper adherence and stabilization prior to analysis.

### Mitostress test (MST)

After 24-hour incubation, media was replaced with 180 µL/well of XF RPMI-1640 medium supplemented with 10 mM glucose, 1 mM pyruvate, 2 mM L-glutamine and was left to stabilize in a non-CO2 37 °C incubator for an additional 1 h prior to running the experiment. Oligomycin targeting a final concentration of 1.5 µM, FCCP targeting a final concentration of 1.0 ~ 2.0 µM, and Rotenone/Antimycin A targeting a final concentration of 0.5 µM were added to ports A, B, and C, respectively. Final measurement values were normalized to the relative Hoechst 33,342 signals normalized to each scrambled cell line.

### Glycolysis stress test (GST)

Following the initial 24-hour incubation, the medium was replaced with 180 µL/well of XF RPMI-1640 medium supplemented with 2 mM L-glutamine. Cells were then incubated for approximately one hour at 37 °C without CO₂ to allow metabolic stabilization before measurements. Glycolytic stress was assessed by sequential injections of 10 mM glucose (port A), 1 µM oligomycin (port B), and 50 mM 2-deoxyglucose (2-DG, port C). Final measurements were normalized to Hoechst 33,342 signals and are presented relative to their respective scrambled control cell lines.

### Metabolite-deprived growth test

The scrambled control and ME2kd cell lines were seeded in 48-well plates at optimized initial densities: MDA-MB-468, 10,000 cells/well; HCC70, 3,500 cells/well; HCC1806, 4,500 cells/well; Hs578T, 4,500 cells/well; BT-20, 9,000 cells/well and MCF10a, 7,000 cells/well. Cells were incubated overnight at 37 °C with 5% CO₂ to facilitate attachment and stabilization. Media was replaced with one of the following experimental conditions: complete RPMI-1640 containing 10 mM glucose (control medium), RPMI-1640 lacking serine and glycine, RPMI-1640 with glucose replaced by 10 mM galactose, or RPMI-1640 lacking serine and glycine with glucose replaced by 10 mM galactose. 10 mM galactose was substituted with 3 mM glucose for Hs578T and HCC1806, which was the minimum concentration required to sustain growth in these cell lines. Each media condition was supplemented with 10% dialyzed FBS (dFBS), 100 U/mL Pen-Strep, and 125 ng/mL doxycycline. Media for MCF10a was additionally supplemented with 0.02 µg/mL EGF, 0.5 µg/mL hydrocortisone, 0.1 µg/mL cholera toxin, and 10 µg/mL insulin which are required for their growth. The medium was refreshed every 24 h.

Cell growth was monitored using an Agilent BioTek BioSpa 8 Automated Incubator and Cytation Cell Imaging Reader (Agilent, Santa Clara, CA, USA), capturing images at 6-hour intervals. The cell counts were normalized relative to the initial cell number seeded in each well. For each cell line and experiment, the final growth points were obtained when one of the groups reached confluence (~ 4–7 days). Endpoint fold-changes were then normalized within genotype: for scrambled and ME2kd cells, deprivation conditions were divided by that genotype’s complete-media control measured on the same plate.

### Malic enzyme 2 and malate-aspartate shuttle co-knockdown growth test

Scrambled control and ME2kd cell lines were seeded in 48-well plates at optimized densities as follows: MDA-MB-468, 10,000 cells/well; HCC70, 3,500 cells/well; HCC1806, 4,500 cells/well; Hs578T 4,500 cells/well; BT-20, 9,000 cells/well; and MCF10a, 7,000 cells/well. Cells were incubated overnight at 37 °C with 5% CO₂ to allow for attachment and stabilization.

Following overnight stabilization, the cells were transfected with siRNA targeting SLC25A13, the major transporter in the MAS (MAS knockdown, MASkd) or negative control siRNA purchased from Integrated DNA Technologies (Coralville, IA, USA). Transfections were performed in 48-well plates using Opti-MEM and Lipofectamine RNAiMAX reagent (Thermo Fisher Scientific), utilizing half of the manufacturer’s recommended volume adjusted appropriately for the 48-well plate conditions. Transfection medium was replaced after 24 h with fresh RPMI-1640 medium supplemented with 100 U/mL penicillin-streptomycin, 10% dialyzed fetal bovine serum (dFBS), and 125 ng/mL doxycycline.

Cell growth was continuously monitored and imaged using an Agilent BioTek BioSpa 8 Automated Incubator paired with a Cytation Cell Imaging Reader (Agilent, Santa Clara, CA, USA), capturing images at 6-hour intervals. For each cell line and experiment, the final growth points were obtained when one of the groups reached confluence (~ 4–9 days). Cell counts from these images were initially normalized to the starting cell number per well, and subsequently to the counts obtained from scrambled control cells transfected with negative control siRNA under identical experimental conditions.

###  Expression and purification of ME2

An ME2 construct comprising amino acids 19–584 (UniProt: P23368) incorporating a C-terminal hexa-histidine tag, was custom synthesized and cloned into a pProEX-HTb plasmid (Genscript) that was transformed into *Escherichia coli* BL21 (LOBSTR) cells. *E. coli* cells were grown at 37 °C in 2YT medium supplemented with 100 µg mL^−1^ ampicillin and with shaking at 180 rpm, to an OD_600_ of 0.8 before incubation at 18 °C and induction with the addition of 0.25 mM isopropyl β-D-1-thiogalactoyranoside (IPTG). The cells were harvested by centrifugation at 4,000 × g at 4 °C for 30 min and were resuspended in lysis buffer comprising 30 mM Tris-HCl pH 7.4, 500 mM NaCl, 10 mM MgCl_2_, 15 mM sodium imidazole, 2 mM β-mercaptoethanol, supplemented with 10 mg/mL lysozyme, 1 mg/mL DNase1, 1 mg/mL RNase H, and EDTA-free protease inhibitor mix (Roche). Cells were mechanically lysed at 18 KPSI using a Microfluidizer M-110P instrument (Brinkmann Instruments), and the lysate centrifuged at 18,000 × g for 30 min at 4 °C. The supernatant was membrane filtered (1.2 μm then 0.45 μm, Millipore) and loaded onto a 5-mL Ni-NTA column (Protino) charged with Ni^2+^ ions and equilibrated with lysis buffer. The column was washed with lysis buffer supplemented with 50 mM imidazole until baseline absorbance was reached. The protein was eluted using a 50–400 mM imidazole gradient over 50 min, and the protein fractions were pooled and concentrated to 1 mL volume. The sample was further purified by size-exclusion chromatography (SEC) using a Superdex 200 10/300 increase column (Cytiva) equilibrated with SEC buffer comprising 30 mM Tris-HCl (pH 7.4), 500 mM NaCl, 10 mM MgCl2, and 2 mM β-mercaptoethanol. Fractions containing ME2 protein identified by SDS-PAGE analysis and western blotting were pooled and concentrated to 20 mg mL^−1^ and were finally flash-cooled in liquid nitrogen for later use. The identity of the ME2 protein was confirmed using mass spectrometry (UoA Mass Spectrometry Center).

### X-ray crystallography

The ME2/NAD^+^ complex was crystallized using sitting drop vapor diffusion in Swiss 2 drop 96 well plates. A 0.20 µL volume of protein (in 30 mM Tris-HCl pH 7.4, 500 mM NaCl, 10 mM MgCl_2_, 2 mM β-mercaptoethanol) was mixed with 0.20 µL of crystallization solution and placed over a 50 µL volume of crystallization solution before sealing. Crystals were grown at 291 K and appeared within four weeks. Crystalline rods were initially observed in Pact condition C4 (Molecular Dimensions) and JSCG + condition B3 (Molecular Dimensions). The microseed matrix screening protocol produced data quality crystals using initial hits as seed stocks [24]. The seed stock was prepared using crystals from the Pact condition C4 (0.1 M PCTP at pH 7.0, 25% w/v PEG 1500), which yielded singular plates in all subsequent crystallization experiments.

Diffraction quality crystals of ME2/NAD^+^ were prepared as above, but using a 0.20 µL volume of protein mixed with 0.25 µL of crystallization solution (0.1 M Bis-Tris, 0.1 M ammonium tartrate, and 20% (w/v) PEG 3350) and 0.05 µL of ME2 crystal seed stock. ME2/NAD/NPD-389 was crystallized identically using the same micro-seeding matrix screening stocks, with the addition of 1 mM NPD-389 (30 mM DMSO stock) to the protein solution and using a crystallization solution comprising 0.1 M SPG (succinic acid, sodium phosphate monobasic monohydrate, glycine) and 25% (w/v) PEG 1500 (Pact A3; Molecular Dimensions). Crystals were cryoprotected in crystallization solution supplemented with 35% (v/v) ethylene glycol, mounted in nylon loops, and were flash cooled in liquid nitrogen for transportation to the Australian Synchrotron.

X-ray diffraction data were collected on the Australian Synchrotron MX2 beamline and processed using XDS and AIMLESS [25, 26]. Structures were solved by molecular replacement using MOLREP or PHASER, and PDB coordinates 8W24 (ME2/NAD/NPD) or 9AYI (ME2/NAD) [27, 28]. Iterative model building and refinement used COOT, REFMAC, and Phenix-refine [29–31]. The structures were validated and deposited in the Protein Data Bank [32]. Data collection and refinement statistics are presented in Tables S1 and S2.

### Recombinant ME2 activity assay

ME2 activity was measured by NADH generation at 340 nm at 37 °C using a Tecan Spark plate reader. The enzyme reaction mixture comprised 100 nM recombinant ME2, 10.0 mM malate, 5.0 mM fumarate, 10 mM MgCl_2_, 1 mM TCEP and 50 mM Tris-HCl, pH 7.4. Initial rates were measured following the addition of 1.0 mM NAD^+^, were normalized to NAD^+^ generation, and fitted to a Michaelis-Menten model. Morrison *K*_i_ plots were produced with activity normalized to an uninhibited control experiment. Data analysis used the Prism 10 software (GraphPad Software).

## Results

### ME2 negatively correlates with genes involved in de Novo Serine synthesis

**Fig. 1:**
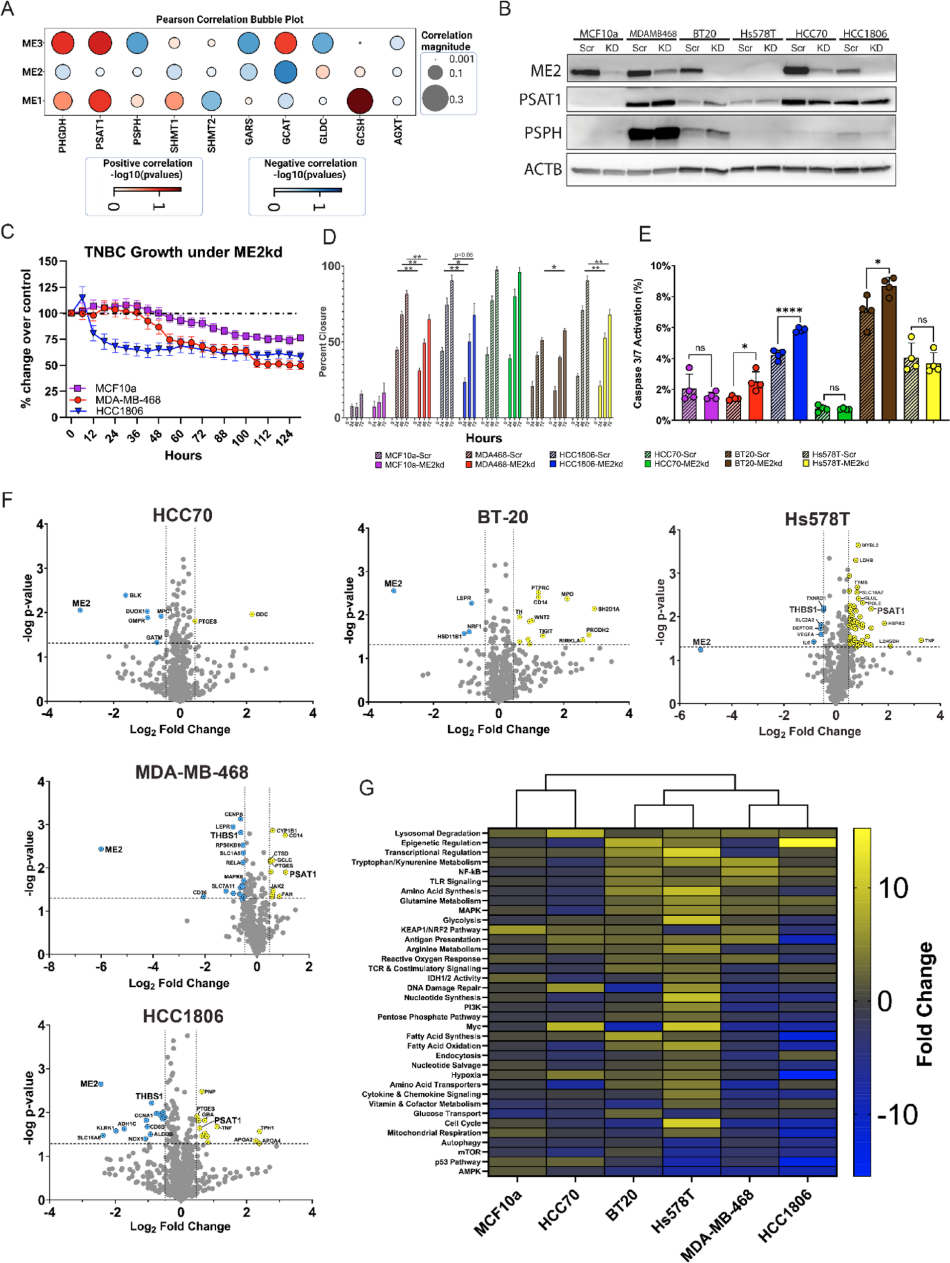
Effect of ME2 knockdown on protein expression and cell growth in TNBC cell lines. **A** Bubble plot showing Pearson correlation of malic enzyme paralog expression (ME1-3) with key serine/glycine pathway genes in TNBC samples. Bubble size indicates correlation strength; red denotes positive correlation, blue denotes negative. Bubble color intensity represents statistical significance (–log10(p-value)). **B** Western blot of ME2, PSAT1, PSPH, and ACTB (loading control) in control (MCF10a) and TNBC cell lines (MDA-MB-468, BT-20, Hs578T, HCC70, HCC1806) under scrambled (Scr) or ME2 knockdown (KD). Representative blot shown (n=3). **C** Cell growth (% change) after ME2 siRNA transfection. Lines with largest growth changes shown; symbols/colors represent different cell lines. Data shown as mean ± SEM (n=3). **D **Donut assay results from listed cell lines; percent closure was calculated with the wound closure tool (ImageJ). Significance determined with two-tailed t-test. **, p < 0.01; ***, p < 0.001. (N=3) **E** Scrambled or ME2-knockdown cells were grown for 3 days in the presence of doxycycline and caspase 3/7 activation was quantified with CellEvent Caspase 3/7 Detection Reagent. Statistical significance was determined with two-tailed t-test. *, p < 0.05; ****, p < 0.0001; ns, not significant (N=3 per group). **F** NanoString Metabolic Pathway volcano plots show gene expression changes (ME2 siRNA vs. control) in indicated cell lines. Axes represent log2 fold-change (x) and -log10 p-value (y). Blue: downregulated; yellow: upregulated; gray: non-significant. Vertical lines indicate ±0.5 log2 fold-change; horizontal line shows significance cutoff (p<0.05) (N=2 per group). **G** Heatmap of gene set analysis (MCF10a and TNBC lines) using Nanostring annotations (nSolver). Hierarchical clustering by Clustergrammer on global significance scores

**Fig. 2:**
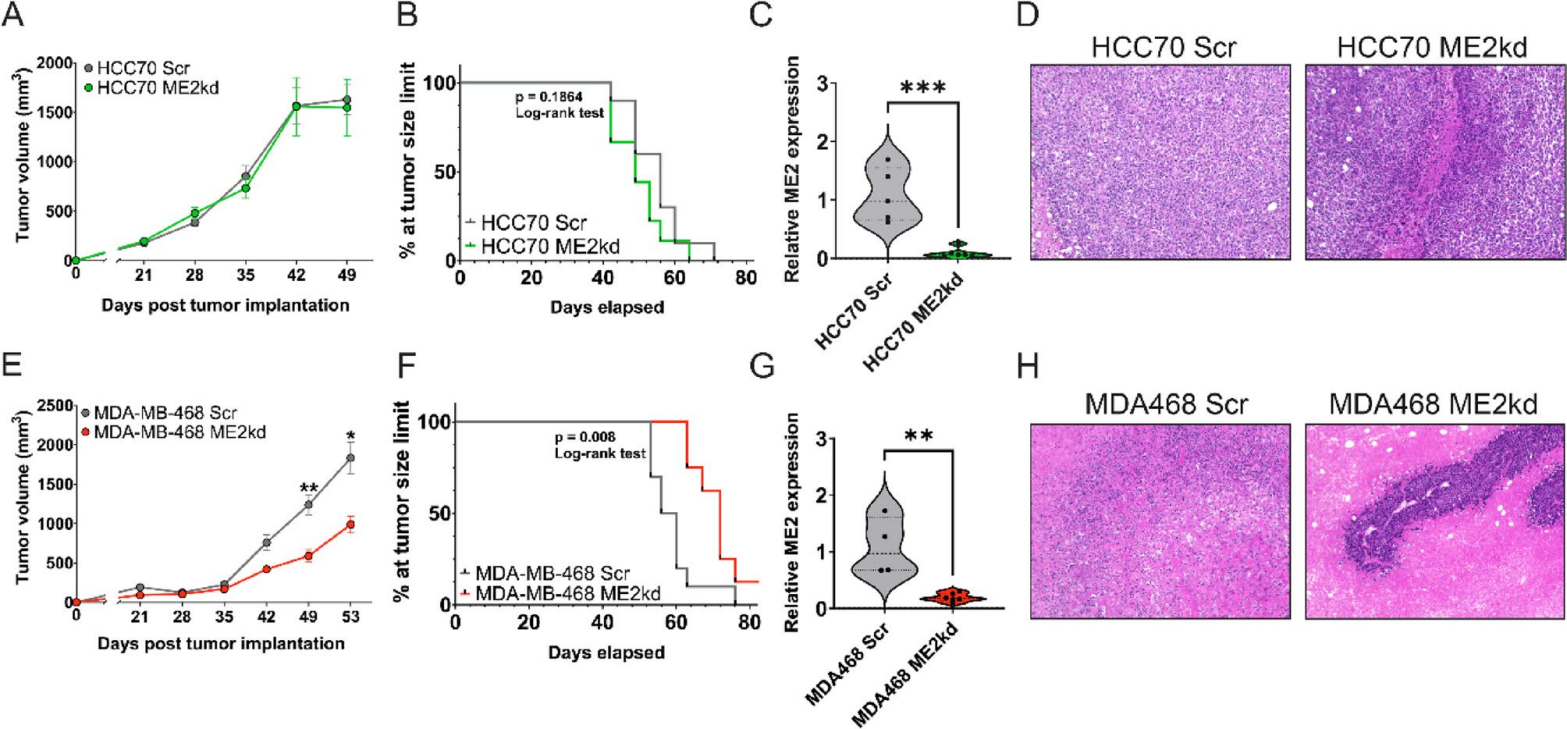
Tumor growth analysis in nude mice implanted with HCC70 (top) or MDA-MB-468 (bottom) cells. (A, E) HCC70 (1.0 x 10^6; A) or MDA-MB-468 cells (1.5 x 10^6; E) transduced with scrambled (Scr) or ME2-targeting shRNA (ME2kd) were transplanted into female nude mice. MDA-MB-468 ME2kd tumors grew significantly slower compared to controls; no growth difference was observed in HCC70. Data represent mean ± SEM (N=10 per group). Statistical significance by two-way ANOVA with Tukey’s test: * p<0.05; ** p<0.01. (B, F) Kaplan-Meier survival curves for mice with HCC70 (B) and MDA-MB-468 (F) tumors. Survival differences analyzed by log-rank (Mantel-Cox) test (N=10 per group). (C, G) Fold change in ME2 expression in HCC70 (C) and MDA-MB-468 (G) tumors quantified by qPCR. Data represent mean ± SEM (Scr, n=5; ME2kd, n=6). Significance by unpaired t-test: **, p<0.01; *** p<0.001. (D, H) Representative 20x H&E images of HCC70 (D) and MDA-MB-468 (H) tumor sections

Previous studies have demonstrated that TNBC cells upregulate genes involved in de novo serine synthesis to sustain their rapid growth [33]. Moreover, the lack of clear therapeutic targets in TNBC has led to the exploration of alternatives that inhibit metabolic enzymes such as malic enzyme 2 (ME2). Therefore, we analyzed TNBC datasets to determine the correlation between ME2 and genes affiliated with serine metabolic pathways [34]. We found that ME2 was negatively correlated with phosphoglycerate dehydrogenase (PHGDH), phosphoserine aminotransferase 1 (PSAT1), and phosphoserine phosphatase (PSPH), which are key enzymes in de novo serine synthesis (Fig. 1A). This lead us to hypothesize that inhibited ME2 expression would create collateral lethality in cells which rely on serine synthesis, due to the inverse correlation that we observed in Fig. 2A. Additionally, we observed a negative correlation between ME2 and other genes downstream of serine metabolism, including Serine Hydroxy methyltransferase 1 and 2 (SHMT1 and SHMT2), Glycyl-tRNA Synthetase (GARS) and Glycine C-Acetyltransferase (GCAT). Notably, the other malic enzyme paralogs, ME1 and ME3, were mostly positively correlated with most genes in the de novo serine synthesis pathway, as well as other metabolic enzymes (Fig. S1). This suggested that their inhibition would be metabolically compensated, thus leading us to pursue ME2 inhibition instead.

### Suppression of ME2 inhibits growth and migration in some TNBC cell lines

Based on our initial analysis, we hypothesized that ME2 inhibition affects de novo serine synthesis and cellular growth rates. ME2 protein expression was knocked down by ME2-shRNA introduced via lentiviral vectors; in total, 6 doxycycline-inducible ME2-targeting shRNA (Horizon Biosciences) were tested in each cell line to determine which had the highest efficacy. We empirically determined that reducing the concentration of doxycycline from the standard (2ug/mL) down to 0.125ug/mL induced knockdown of ME2 via shRNA expression without effecting mitochondrial function (Fig. S2C). While ME2 expression was effectively knocked down, ME1 was unchanged (Fig. S2A, B) and ME3 expression was not detectable (data not shown). In every cell line tested, removal of doxycycline from the media restored ME2 expression to endogenous levels (data not shown). ME2kd resulted in variable changes in phosphoserine aminotransferase 1 (PSAT1) and phosphoserine phosphatase (PSPH) protein expression between TNBC cell lines (Fig. 1B). Growth assays revealed a decrease in proliferation of 50% for MDA-MB-468 and HCC1806 cell lines (Fig. 1C). In contrast, the proliferation of the non-tumorigenic MCF10a cells was reduced by only 25%. The remaining TNBC cell lines did not exhibit significant growth inhibition (data not shown) compared to MCF10a cells, suggesting that ME2 dependency varies among TNBC models.

In addition to its effect on proliferation, ME2kd impaired the migratory capacity of TNBC cells. Donut assays revealed a reduction in the migratory potential of MDA-MB-468 and HCC1806 cells following ME2kd, with MCF10a remaining unaffected (Fig.1D). Although Hs578T was not growth-restricted under ME2kd conditions, the migratory capacity was significantly affected. Interestingly, BT-20 cells showed increased migration at 72 h. The impaired migration observed in MDA-MB-468, HCC1806, and Hs578T cells suggests that ME2 may play a role in metastatic behavior. The differential response in migration further supports the notion of variable reliance on ME2 in highly heterogeneous TNBC tumors.

### The metabolic transcriptome is dysregulated under ME2 knockdown

To investigate the effects of ME2kd on the metabolic transcriptome, we used the NanoString Metabolic Pathways Panel, which examined the expression of ~ 750 metabolic genes. ME2 silencing was achieved per cell line with a panel of 3 different siRNA targeting ME2; the siRNA with the highest efficacy was chosen for each cell line independently. As expected, ME2kd resulted in extensive ME2 downregulation across all tested cell lines. We observed substantial changes in metabolic genes, including PSAT1, which was upregulated in MDA-MB-468, HCC1806, and Hs578T cells, but not in HCC70 or BT-20 cells (Fig. 1F). Gene set analysis with Nanostring pathways revealed a high degree of similarity between MDA-MB-468 and HCC1806 cells, both of which were growth-restricted under ME2kd (Fig. 1G). Overall, ME2kd resulted in variable responses to the expression of other metabolic genes.

### Tumors derived from ME2kd MDA-MB-468 cells grow slower in nude mice

We assessed the growth of HCC70 and MDA-MB-468 cells under ME2kd within the context of whole-body metabolism using in vivo xenograft studies, comparing ME2kd to the scrambled control. These two cell lines were selected for their opposing phenotypes in vitro under ME2kd; HCC70 was not growth-restricted, whereas MDA-MB-468 grew 50% slower. Tumors derived from MDA-MB-468 cells under ME2kd exhibited slower growth than the scrambled control, while the HCC70-ME2kd tumors were not restricted (Fig. 2A, E). MDA-MB-468-ME2kd tumors grew linearly, in contrast to the exponential growth of scrambled controls at 35 days post-implantation. Kaplan-Meier survival curves showed an extension in survival time for mice bearing MDA-MB-468-ME2kd tumors compared to those bearing scrambled shRNA (*p* = 0.008 (log-rank test) (Fig. 2F). There was no difference in survival in the HCC70 group (*p* = 0.1864) (Fig. 2B). Knockdown of ME2 expression was confirmed in both HCC70 and MDA-MB-468 derived tumors after dissection by qPCR (*p* < 0.001, *p* < 0.01; Fig. 2C, G). H&E staining of tumor sections (Fig. 2D, H) showed that ME2kd tumors were more compact, suggesting a possible defect in their metastatic potential.

### Malic Enzyme 2 Knockdown Induces a Negative Correlation Between Mitochondrial Respiration and Glycolysis

Due to ME2’s role in the mitochondria, we next evaluated mitochondrial respiration and glycolytic function using Seahorse analysis via the mitochondrial stress test (MST) and glycolysis stress test (GST), respectively.

**Fig. 3:**
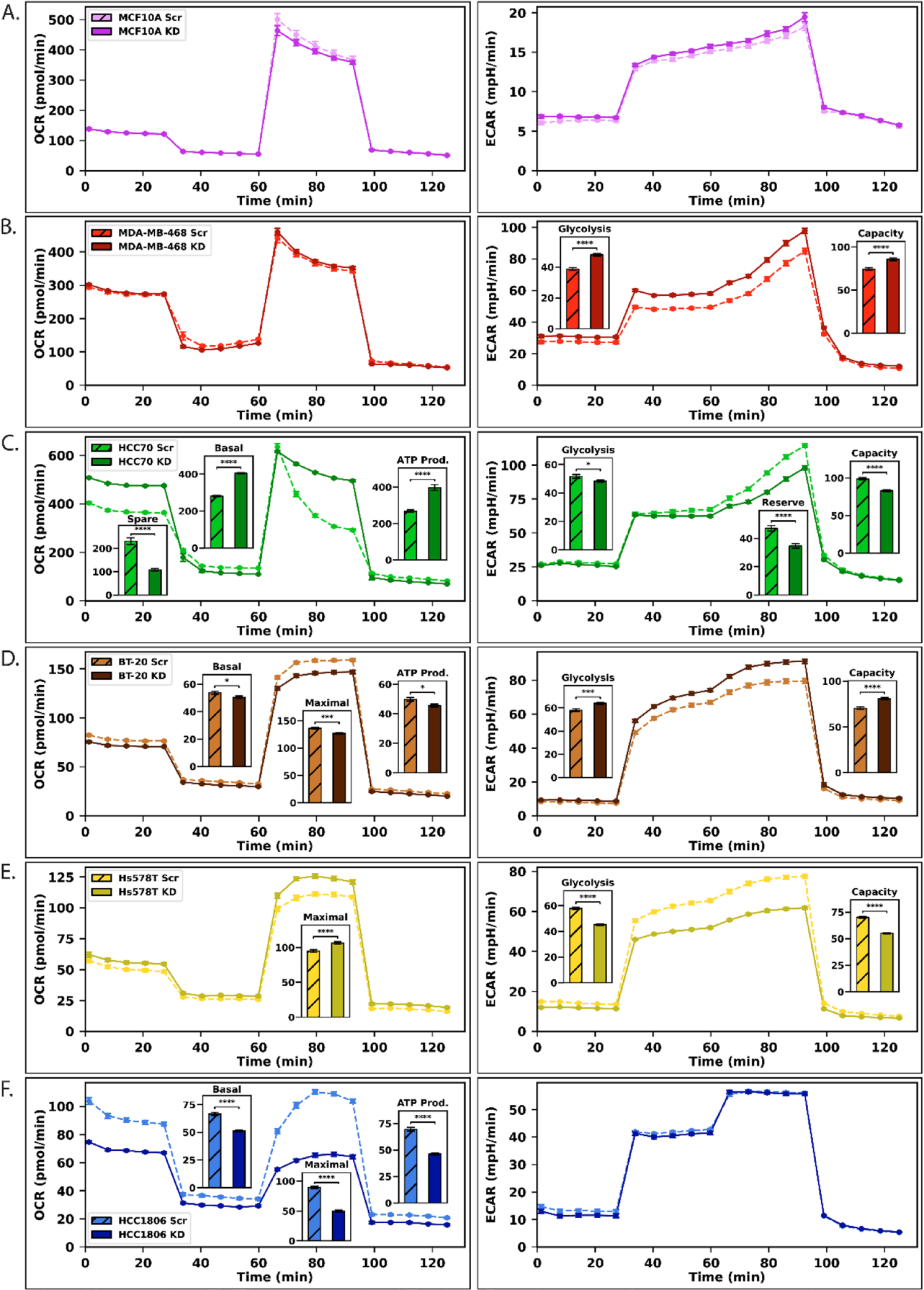
Seahorse Mito Stress Test OCR (Left) and Glycolysis Stress Test ECAR (Right) of scrambled (Scr) and ME2kd (KD) of the 6 cell lines. **A** Seahorse MST (Left) (Scr: n = 11, ME2kd: n = 12) and GST (Right) (Scr: n = 22, ME2kd: n = 24) of MCF10A. **B** Seahorse MST (Left) (Scr: n = 16, ME2kd: n = 16) and GST (Right) (Scr: n = 22, ME2kd: n = 24) of MDA-MB-468. **C** Seahorse MST (Left) (Scr: n = 22, ME2kd: n = 24) and GST (Right) (Scr: n = 22, ME2kd: n = 24) of HCC70. **D** Seahorse MST (Left) (Scr: n = 22, ME2kd: n = 24) and GST (Right) (Scr: n = 22, ME2kd: n = 24) of BT-20. **E** Seahorse MST (Left) (Scr: n = 12, ME2kd: n = 12) and GST (Right) (Scr: n = 22, ME2kd: n = 24) of Hs578T. **F** Seahorse MST (Left) (Scr: n = 22, ME2kd: n = 24) and GST (Right) (Scr: n = 30, ME2kd: n = 32) of HCC1806. Inset of MST data include data for Spare Capacity, Basal Respiration, Maximal Respiration, and ATP Production. Inset of GST data include data for Glycolysis, Glycolytic Reserve, and Glycolytic Capacity. Data normalized with Hoechst-33342 relative to the control. Error bars indicate SEM

The effects on both MST and GST of ME2kd varied widely across TNBC models. No statistical differences in MST were observed between the scrambled and MDA-MB-468-ME2kd lines (Fig. 3B). Most changes in MDA-MB-468-ME2kd cells were observed with GST. Changes were observed in glycolysis (+ 24% in ME2kd, *p* < 0.001) and glycolytic capacity (+ 16% in ME2kd, *p* < 0.001).

HCC70 cells showed enhanced mitochondrial respiration but reduced glycolytic function (Fig.3C). MST results revealed increases in basal respiration (+ 44% in ME2kd, *p* < 0.001) and mitochondrial ATP production (+ 48% in ME2kd, *p* < 0.001) and a decrease in spare capacity (−53% in ME2kd, *p* < 0.001). GST showed a minor decrease in glycolysis (−7% in ME2kd, *p* = 0.04) and a moderate decrease in glycolytic capacity (−16% in ME2kd, *p* < 0.001). Notably, HCC70 also showed a decrease in glycolytic reserve (−26% in ME2kd, *p* < 0.001).

In contrast, mitochondrial respiration decreased, whereas glycolytic function increased with ME2kd in BT-20 cells (Fig. 3D). MST results showed moderate decrease in basal respiration (−6% in ME2kd, *p* = 0.04), maximal respiration (−8% in ME2kd, *p* = 0.01), and ATP production (−7% in ME2kd, *p* < 0.001). GST results showed moderate increase in glycolysis (+ 10% in ME2kd, *p* < 0.001) and glycolytic reserve (+ 15% in ME2kd, *p* < 0.001).

Hs578T-ME2kd cells exhibited moderate changes in mitochondrial activity and significant changes in glycolytic function (Fig. 3E). MST results showed a moderate increase in maximal respiration (+ 12% in ME2kd, *p* < 0.001). GST results showed major decreases in glycolysis (−22% in ME2kd, *p* < 0.001) and glycolytic capacity (−21% in ME2kd, *p* < 0.001).

ME2kd caused major changes in HCC1806 in mitochondrial function but exhibited no statistically significant changes in glycolysis (Fig. 3F). MST results showed decreases in basal respiration (−23% in ME2kd, *p* < 0.001), maximal respiration (−34% in ME2kd, *p* < 0.001), and ATP production (−44% in ME2kd, *p* < 0.001). In contrast, the GST results showed no difference between the scrambled and ME2kd groups. No differences were observed between the normal-like MCF10a scrambled and MCF10a-ME2kd cells, either in terms of mitochondrial or glycolytic function (Fig. 3A).

### Malic enzyme 2 knockdown causes large changes to malate and its related metabolites

**Fig. 4:**
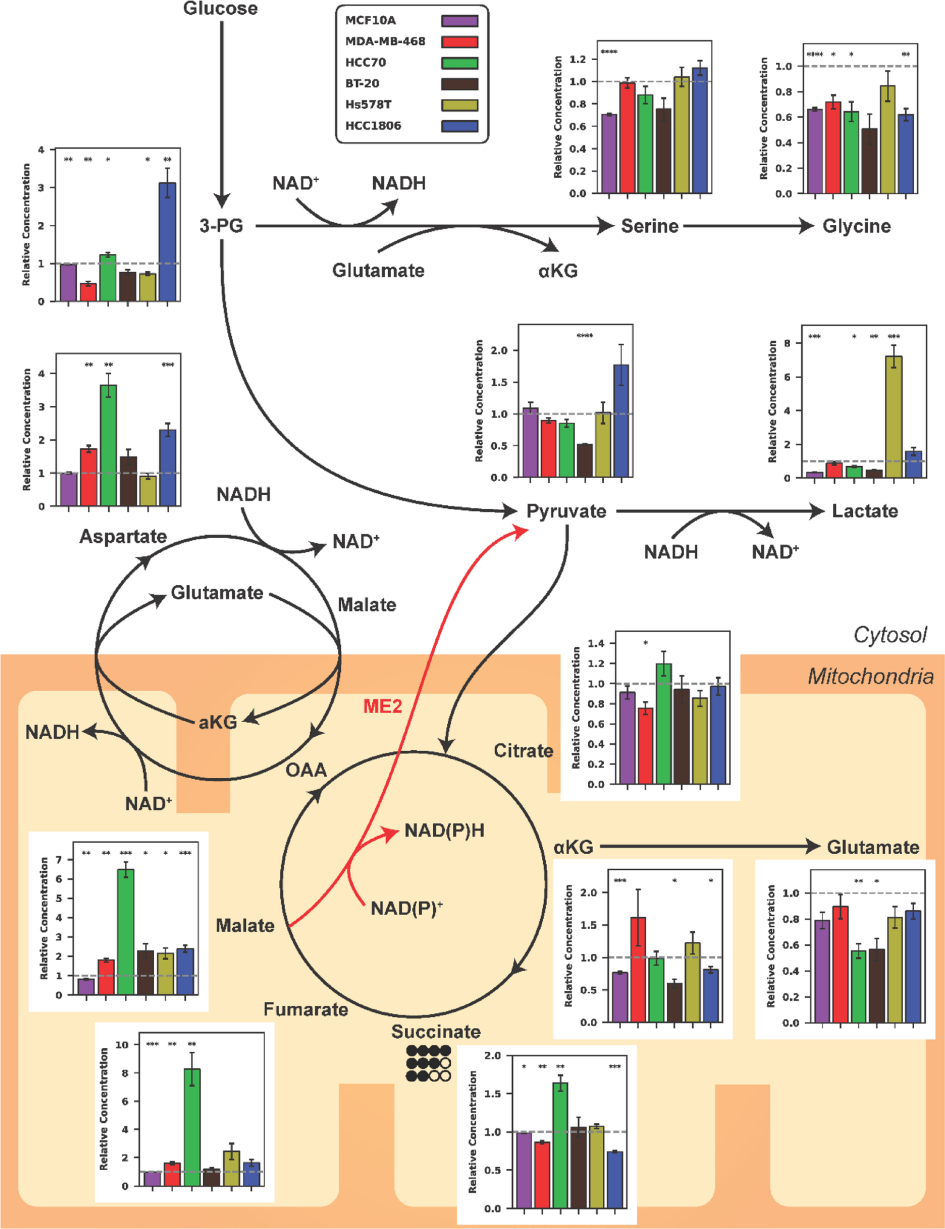
GC-MS metabolite concentrations of central carbon metabolism under complete media conditions. Bars represent metabolite levels in ME2kd cells normalized to scrambled controls (each n=3). Data normalized to norvaline internal standard; error bars represent error propagation. Circles indicate TCA cycle (bottom mitochondria) and MAS pathway (left, mitochondria-cytosol interface)

The observed functional changes in the mitochondria led us to investigate metabolite levels. We used GC-MS to quantify the concentration of malate, the substrate of ME2, and its relevant metabolites, as depicted in Fig. 4.

Malate levels increased in all TNBC lines, from a 1.80-fold increase in MDA-MB-468 cells (*p* < 0.01) to a 6.48-fold increase in HCC70 cells (*p* < 0.001). In contrast, the non-cancerous MCF10a cell line exhibited a 1.24-fold decrease. Most TNBC cell lines showed negligible change in fumarate concentration following ME2 knockdown. However, HCC70 exhibited a ~ 8-fold increase in fumarate. Fumarate is known to allosterically activate ME2 [35]. Thus, its accumulation may have partially compensated for the loss in expression of the enzyme. This could help explain the lack of significant change observed in growth in vitro and in vivo for this cell line (Fig. 2A-D).

Only BT-20 and HCC1806 showed notable shifts in pyruvate, with BT-20 decreasing 1.92-fold (*p* < 0.001) and HCC1806 increasing 1.77-fold (*p* = 0.06). However, lactate changed significantly in most TNBC lines, suggesting a potential flux redirection between lactate and pyruvate. Lactate levels decreased in HCC70 (1.49-fold, *p* = 0.02) and BT-20 (2.23-fold, *p* < 0.01), but increased sharply in Hs578T cells (7.21-fold, *p* < 0.001) and modestly in HCC1806 (1.57-fold, *p* = 0.05).

Glutamate levels declined across TNBC cell lines, especially in HCC70 (1.81-fold decrease, *p* < 0.01) and BT-20 (1.77-fold decrease, *p* = 0.02). Glutamine utilization as an alternative energy source is a common phenomenon in cancer; however, ^13^C_5_-glutamine labeling revealed no significant changes in either oxidative or reductive TCA cycle activity (Figure S7).

Conversely, aspartate levels increased notably in MDA-MB-468 (1.73-fold, *p* < 0.01), HCC70 (3.65-fold, *p* < 0.01), and HCC1806 (2.3-fold, *p* < 0.001). Serine concentrations remained mostly unchanged, except for a 1.51-fold decrease in MCF10a cells (*p* < 0.001). Glycine generally decreased among the TNBC lines, ranging from a 1.39- to 1.97-fold decrease, and reached significance in MDA-MB-468 (*p* = 0.03) and HCC1806 (*p* < 0.01).

Given the consistency between mean enrichment and mass isotopologue distribution (MID) data, we summarized labeling results with mean enrichment data. Detailed MIDs are available in Supplementary Figures S5 and S6.

### Serine/Glycine deprivation highlights Cell-Specific TCA perturbations with ME2 knockdown

**Fig. 5:**
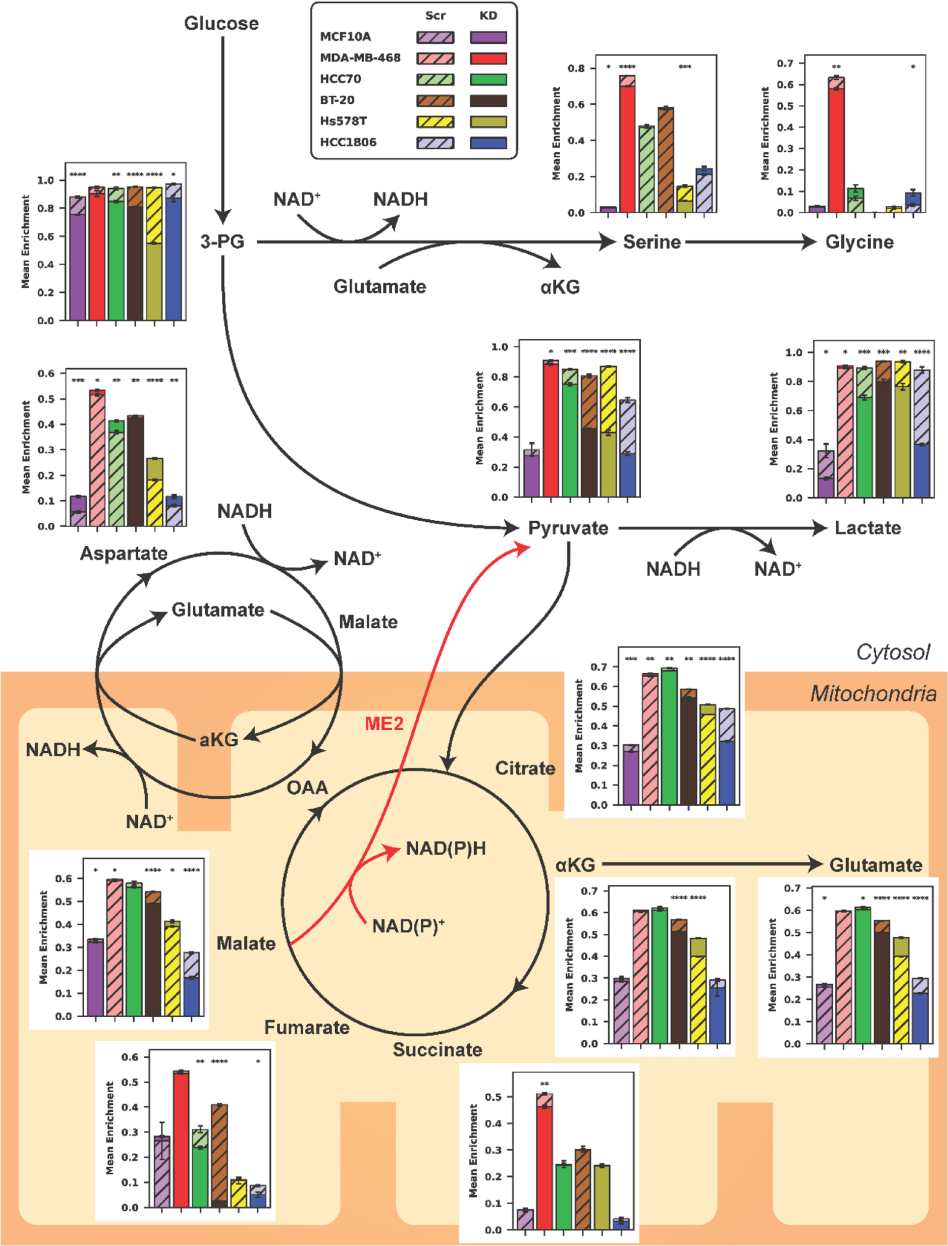
GC-MS mean enrichment of central carbon metabolism under serine/glycine-deprived conditions. Bars represent metabolite enrichment ratios for scrambled or ME2kd cell lines (each n=3). Data normalized to norvaline internal standard; error bars represent SEM. Circles indicate TCA cycle (bottom mitochondria) and MAS pathway (left, mitochondria-cytosol interface)

Building on previous findings linking the loss of ME2 with increased expression of de novo serine synthesis enzymes (Fig. 1F), we conducted a glucose-labeled tracing experiment under serine and glycine‐deprived conditions to examine how ME2kd influences malate and serine metabolism (Fig. 5). Restricting serine and glycine triggered distinct TCA metabolic responses in each cell line. MCF10a and MDA-MB-468 showed minimal shifts in TCA labeling after ME2kd, whereas BT-20, Hs578T, and HCC1806 exhibited altered labeling of malate and fumarate. Malate labeling decreased by up to 11% in HCC1806 (*p* < 0.001), but increased by 2% in Hs578T (*p* = 0.02). Fumarate labeling decreased by 7% in HCC70 (*p* < 0.01), 38% in BT-20 (*p* < 0.001), and 4% in HCC1806 (*p* = 0.03).

Pyruvate labeling declined in all TNBC lines yet remained unchanged in MCF10a, ranging from a 2% decrease in MDA-MB-468 (*p* = 0.03) to a 44% decrease in Hs578T (*p* < 0.001). Lactate followed a similar trend, with reductions of up to 51% in HCC1806 (*p* < 0.001). α-Ketoglutarate and glutamate showed minimal changes in MCF10a, MDA-MB-468, and HCC70 cells, while BT-20 and HCC1806 exhibited decreased labeling, and Hs578T showed increased labeling. α-Ketoglutarate labeling increased by 8% in Hs578T (*p* < 0.001) but decreased by 5% in BT-20 (*p* < 0.001). Likewise, glutamate labeling increased by 8% in Hs578T (*p* < 0.001), but declined in other TNBC lines. Aspartate displayed an opposite pattern in most cases, rising 4% in HCC70 (*p* < 0.01), 9% in Hs578T (*p* < 0.001), and 4% in HCC1806 (*p* < 0.01), but decreased slightly (0.9%) in BT-20 (*p* < 0.01).

Only MDA-MB-468 and Hs578T showed significant differences in serine labeling between scrambled and ME2kd cells under serine/glycine-deprived conditions, with reductions of 6% (*p* < 0.001) and 8%, respectively.

Under full-media conditions (Fig. S3), all TNBC lines displayed significant alterations in serine labeling. MDA-MB-468 (6%, *p* < 0.001), HCC70 (2%, *p* < 0.001), and BT-20 (0.8%, *p* < 0.01) cells increased, whereas Hs578T (4%, *p* < 0.001) and HCC1806 (5%, *p* < 0.001) cells decreased. Glycine labeling increased in HCC70 (2%, *p* = 0.01) and BT-20 (0.7%, *p* < 0.01), but declined in Hs578T (2%, *p* < 0.001) and HCC1806 (5%, *p* = 0.06). MCF10a showed no significant changes in serine or glycine labeling under full medium conditions.

### Glucose, Serine, and Glycine-Deprivation Shows Increased Reliance on Glycolysis and De Novo Serine Synthesis in TNBC

**Fig. 6:**
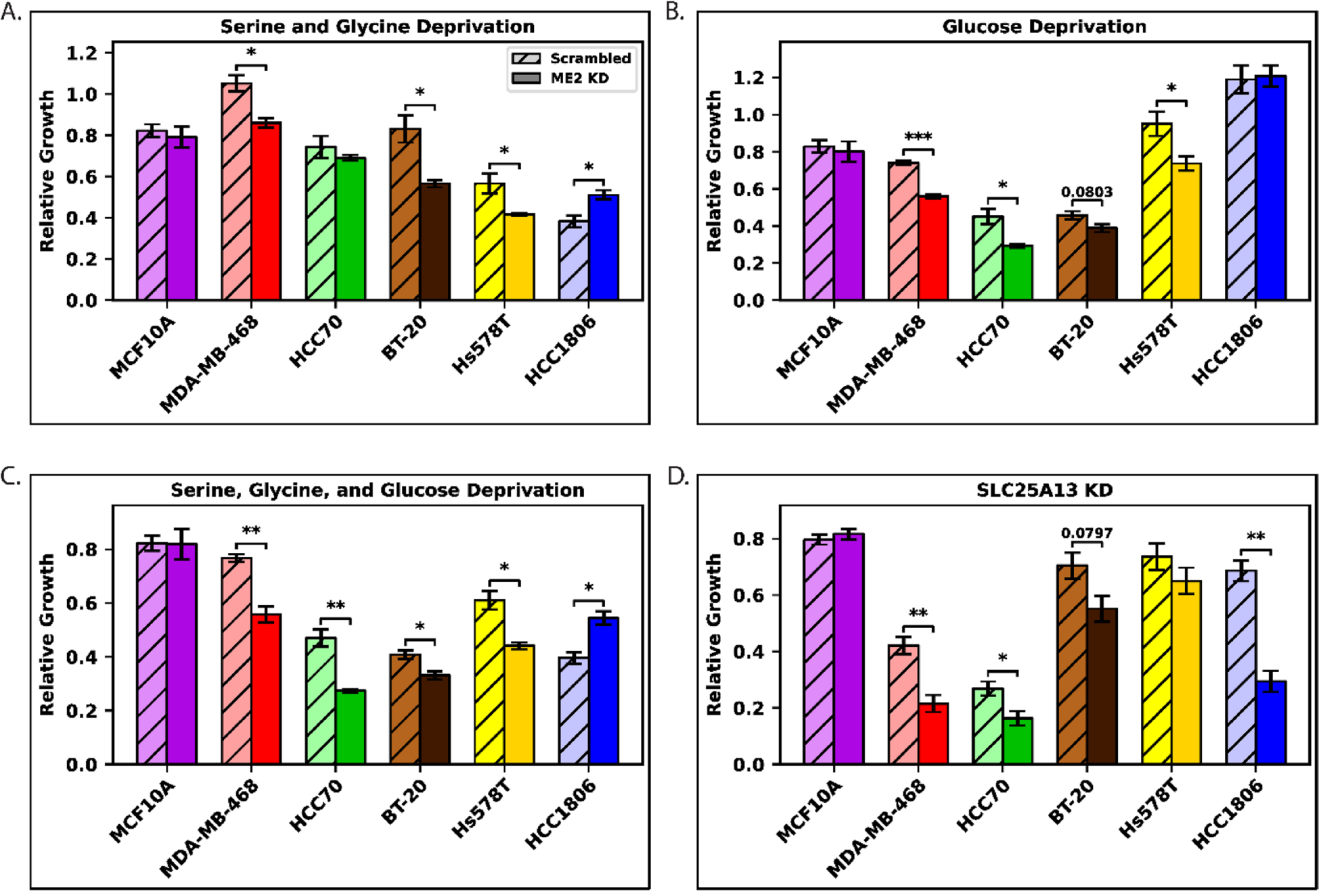
Relative growth (normalized within genotype) under metabolite deprivation and MAS knockdown (MASkd). For all conditions, each bar was normalized to the complete-media control of its own genotype (scrambled or ME2kd). Thus, scrambled cells under deprivation were compared to scrambled cells in complete media, and ME2kd cells under deprivation were compared to ME2kd cells in complete media. **A** Growth under serine/glycine deprivation. **B** Growth under glucose deprivation. **C** Growth under combined serine/glycine/glucose deprivation. **D** Relative growth under MASkd, where cells transfected with MAS siRNA were normalized to their matched non-targeting siRNA controls. Bars represent mean ± error-propagated SEM (each condition/cell line, n = 3)

Based on the GC-MS results, we performed growth tests in medium deprived of serine, glycine, and glucose. We compared scrambled and ME2kd lines grown in media lacking these components using full-media controls as a reference (Fig. 6).

Under serine/glycine deprivation, most TNBC cell lines showed reduced growth, with a minimal impact on MCF10a cells. MDA-MB-468 had a 19% absolute and 18% relative decrease in growth in ME2kd (*p* = 0.01). BT-20 displayed a 27% absolute and 32% relative reduction (*p* = 0.02), and Hs578T displayed a 15% absolute and 27% relative decrease (*p* = 0.04). Notably, HCC1806 exhibited a 13% absolute and 34% relative increase in growth under serine/glycine deprivation (Fig.6A).

Low-glucose conditions produced significant differences in MDA-MB-468 and HCC70 cells, but not in other TNBC lines. MDA-MB-468 showed an 18% absolute and 24% relative decrease in ME2kd vs. scrambled (*p* < 0.001), whereas HCC70 experienced a 16% absolute and 35% relative decrease (*p* = 0.02). BT-20 showed a 7% absolute and 15% relative decrease (*p* = 0.08), while Hs578T showed a 21% absolute and 23% relative decline (*p* = 0.05). MCF10a cells showed no difference (Fig.6B).

When both serine/glycine and glucose levels were depleted, all TNBC lines exhibited significant changes. MDA-MB-468 had a 21% absolute and 27% relative decrease in the ME2kd line (*p* < 0.01), while HCC70 showed a 20% absolute and 42% relative decrease (*p* < 0.01). BT-20 showed a moderate 8% absolute and 19% relative decrease (*p* = 0.02). Hs578T displayed a 17% absolute and 28% relative decline (*p* = 0.01). HCC1806 again showed increased growth of 15% absolute and 37% relative (*p* = 0.01). MCF10a cells remained unaffected under these conditions (Fig. 6C), indicating that metabolic adaptations to ME2kd are specific to TNBC cell lines.

### The Malate-Aspartate Shuttle Works in Concert with Malic Enzyme 2 to Regulate Cell Growth in TNBC

The results of the metabolite deprivation test indicated an interplay between ME2, glycolysis, and serine metabolism. However, further studies were required to elucidate the connections between these metabolic pathways. To this end, we investigated the potential role of the malate-aspartate shuttle (MAS) in this system. MAS was knocked down using siRNA targeting SLC25A13 in the 6 cell lines in both the scrambled and the ME2kd cell lines. The results of the relative growth of each cell line compared to their negative siRNA controls are shown in Fig. 6D.

Most TNBC models showed a significant difference between scrambled and ME2kd lines with malate-aspartate shuttle knockdown (MASkd). MDA-MB-468 exhibited a 20% absolute and 50% relative decrease in growth in the ME2kd cell line as compared to the scrambled cell line (*p* < 0.01). HCC70 saw a 10% absolute and 40% relative decline in growth in the ME2kd line (*p* = 0.04). BT-20 showed a 15% absolute and 22% relative decline in growth comparing the ME2kd line and the scrambled line (*p* = 0.08). HCC1806 exhibited a 40% absolute and 57% relative decrease in growth in the ME2kd line (*p* < 0.01). MCF10a and Hs578T showed no significant difference between the scrambled and ME2kd cell lines (Fig. 6D).

### NPD-389 coordinates the active site metal ion and outcompetes malate binding in ME2

**Fig. 7:**
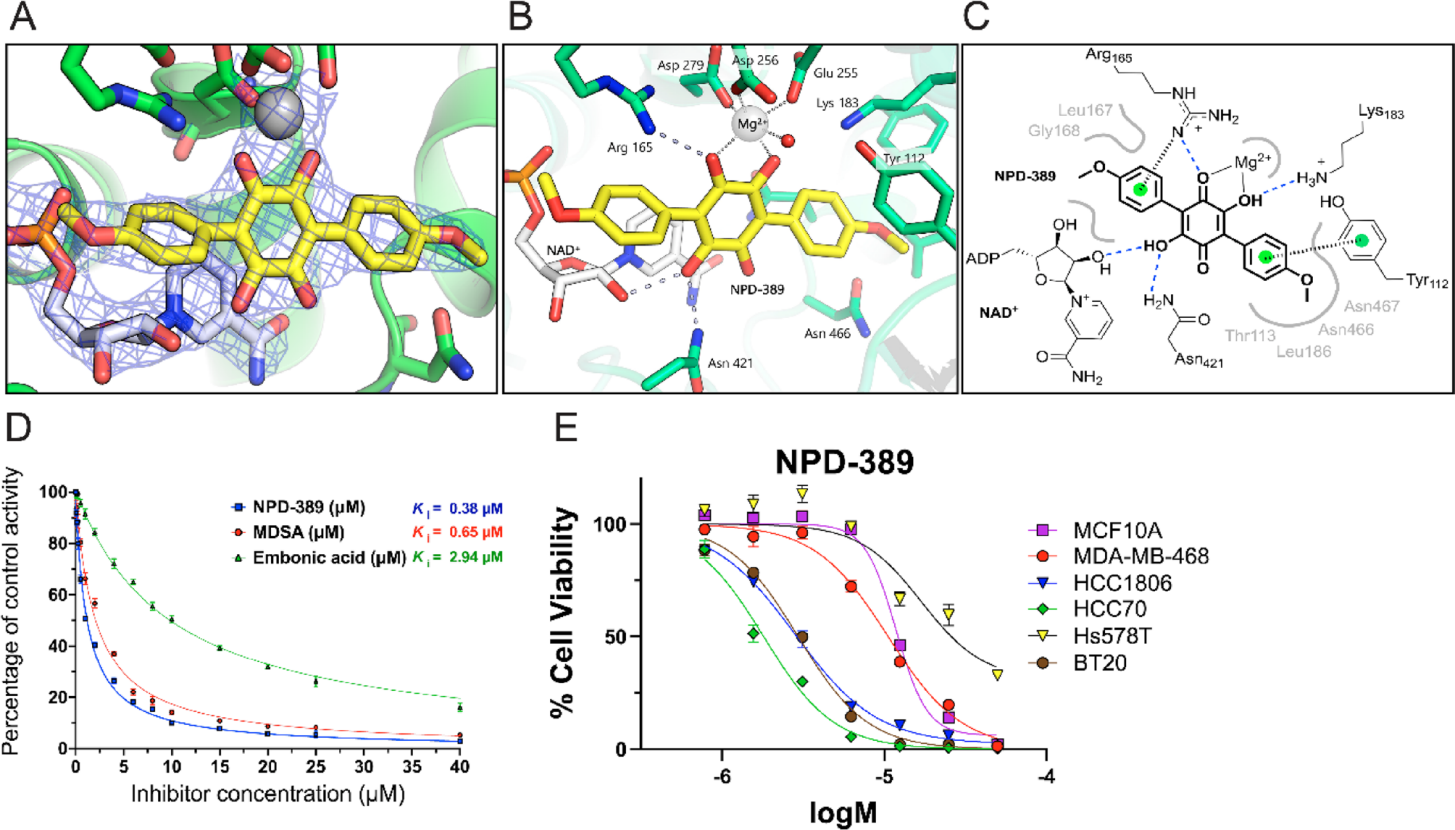
ME2 inhibition and binding by NPD-389. **A** Binding mode of NPD-389 confirmed by visual inspection and electron density (2Fo-Fc omit map). **B **Crystal structure of ME2–NPD-389 complex. NPD-389 (yellow sticks) binds Mg²⁺ (gray sphere) via its central ring, adjacent to NAD cofactor (white sticks). Metal coordination (gray dashed lines) involves a coordinated water molecule (red sphere); additional protein contacts shown as dashed lines. The 4-OMe-substituted phenyl rings project toward solvent (left) or into hydrophobic pocket (right). **C** Close-up of ME2–NPD-389 interactions: metal coordination (solid lines), hydrogen bonds (blue dashed lines), aromatic π interactions (hashed lines to green-ring centroids). Loop with Leu167/Gly168 side chains shown by bold gray line; hydrophobic pocket indicated by gray curve. **D** Recombinant ME2 inhibition by NPD-389, MDSA, and embonic acid (5 mM fumarate present). Morrison Ki plots show normalized activity vs inhibitor concentration (protein 100 nM; cofactor 1 mM; substrate 10 mM). Competitive inhibition by NPD-389 confirmed by substrate variation assays (not shown), consistent with structure and Wen et al., 2014. **E** Dose-response curves for NPD-389 in TNBC lines. Concentrations in log(M); growth response shown for each cell line (n=3)

We solved the structure of ME2/NAD^+^/NPD-389 and ME2/NAD^+^ (holoenzyme) at 2.45 Å and 1.89 Å, respectively. Both structures display the canonical malic enzyme tetrameric assembly, with NAD^+^ located within each active site cavity and buried within the tetramer interface. The ME2/NPD-389 tetramer assembly is produced by crystallographic symmetry from the dimeric crystal structure. The inhibitor is localized within each active site adjacent to an NAD^+^ molecule (Fig. 7A-C).

In each of the two unique active sites, NPD-389 coordinates Mg^2+^ through its central 2,5-dihydroxyl benzoquinone ring, displaying metal-oxygen distances of 2.0 Å. NPD-389 binding is competitive with respect to L-malate, with both molecules sharing the same site and metal coordination (Fig. S9A), which is consistent with the literature [36, 37] and our own enzyme kinetics and inhibition data (Fig.7D). The 4-OMe-substituted phenyl groups of the symmetrical molecule project either into a non-polar pocket within the protein interior or out into the solvent space. Within the non-polar pocket, a small section of the protein centered on Thr113 moves to avoid a clash (Fig. S9B), and a potential weak hydrogen bond is formed between the O-Me group and Asn466. Compared to L-malate binding, the Tyr112 residue, purportedly facilitating proton transfer in catalysis, rotates away from the active site and forms π–π ring-stacking interactions with the buried 4-OMe-substituted phenyl group of NPD-389 (Fig.7B and C; Fig. S9A). Analysis of the more solvent-exposed end of the active site is more complex, with the 4-OMe-substituted phenyl less sterically restrained and with two alternative NAD^+^ conformations observed at different sites of the holoenzyme structure. In chains C and D of the holoenzyme, NAD^+^ displays the same localization and conformation as in the NPD-389 structure, while in chains A and B, the nicotinamide group of NAD^+^ is flipped approximately 180°, forming polar interactions with Arg165 and Asp279 that would clash with the observed inhibitor binding mode. Irrespective of the NAD^+^ configuration, inhibitor binding at this solvent-exposed end induces a conformational rearrangement of the protein

between Glu164 and Gly170 to eliminate a clash with the side chain of Leu167, similar to that observed for L-malate binding (Fig. S). The solvent-exposed 4-OMe-substituted phenyl also forms potential π interactions with the Leu 167 backbone oxygen and the side chain of Arg 165. A detailed illustration of the intermolecular interactions is provided in Fig. 7C.

### Target engagement of ME2 by NPD-389 in isolated mitochondria

A substrate-uncoupler-inhibitor-titration (SUIT) protocol was employed to validate the target engagement of ME2 by NPD-389 in intact mitochondria isolated from F-293 cells. In the absence of NPD-389, the mitochondria exhibited near-maximal respiration in the presence of malate and ADP (Fig. S6A, B). However, NPD-389 inhibited malate-driven respiration in a dose-dependent manner, which was reversed to vehicle control levels upon addition of pyruvate. At concentrations 10-fold and the Ki for ME2, NPD-389 decreased malate respiration versus vehicle control by ~ 50% and 80% without compromising components of the electron transport system.

### NPD-389 inhibits growth in some TNBC models

To evaluate the potential of the known ME2 inhibitor NPD-389 to inhibit the growth of TNBC, we assessed cell viability in TNBC cell lines. NPD-389 (Fig. 7E) evoked varying dose-dependent decreases in the viability of HCC1806, HCC70, and BT-20 compared to MCF10a; MDA-MB-468 was only minimally affected. Notably, the IC_50_ was lower for all TNBC cell lines except for Hs578T compared to the MCF10a control – MCF10a, 11.9µM; MDA-MB-468, 10.5µM; HCC1806, 2.8µM; HCC70, 1.8µM; Hs578T, 17µM; and BT-20, 3µM, indicating that while NPD-389 is not effective enough to serve as a potential drug, structural modifications to the compound would likely improve the efficacy.

## Discussion

ME2 has emerged as an important mediator of metabolic states in cancers, including TNBC, by modulating the production of NAD(P)H and, to a lesser extent, pyruvate. Given the reliance on glutamine for many aggressive cancer subtypes [38] and the capacity to convert glutamine into malate through the TCA cycle, increased expression of malate-processing enzymes such as ME2 is an important adaptation in cancer. In this study, we aimed to determine the contribution of ME2 to TNBC cell growth, migration, and metabolic flux. TCGA analysis revealed a negative correlation between ME2 and de novo serine synthesis gene expression, as well as several genes relating to glycine metabolism (GCAT, GAMT, GNMT, and SHMT1) (Fig. 1A). As glycine, along with glutamate and cysteine, is a component of glutathione (GSH) this may suggest that ME2 expression may also be connected to redox regulation via this pathway. This was supported by our GC-MS data that showed changes in concentration and labelling for glycine and glutamate in Figs. 4 and 5. ME2kd significantly reduced the proliferation of MDA-MB-468 and HCC1806 cells. ME2 depletion led to a striking reduction in migration of MDA-MB-468, HCC1806, and Hs578T cells, indicating that ME2 may enhance metastatic potential, similar to its reported role in melanoma [39]. The differential response in migration, with BT-20 showing increased migration at 72 h, further supports the notion of variable reliance on ME2 in highly heterogeneous TNBC tumors.

The effects on growth and metastatic phenotypic features observed in vitro were consistent with the in vivo behavior of the ME2kd xenografts. MDA-MB-468-ME2kd tumors progressed more slowly than the scrambled controls, particularly at later stages of tumor progression. Importantly, survival analysis showed prolongation of survival of mice bearing MDA-MB-468-ME2kd tumors. However, we observed that ME2 played a relatively minor role in some TNBC models. This diversity in phenotypes may be due to differences in metabolic dependencies or genetic backgrounds. For example, in terms of the TNBC cell lines utilized in the in vivo tests, the MDA-MB-468 cell line exhibits amplification of EGFR and expresses high levels of the protein, while HCC70 does not [40, 41]. The differential dependence on EGFR signaling could be an important factor related to the induction of different cell states by ME2 inhibition, which requires further investigation.

The observed interplay between mitochondrial respiration and glycolysis provides valuable insights into metabolic shifts following ME2kd. Notably, there was an overall inverse trend between the changes in mitochondrial respiration and glycolytic activity (Fig. 3). Cell lines with increased OCR, such as HCC70 and Hs578T, displayed decreased ECAR, whereas cell lines with reduced OCR, such as BT-20 and HCC1806, displayed either increased ECAR (BT-20) or no change (HCC1806). In contrast, MDA-MB-468 cells showed no clear change in OCR but exhibited a significant increase in ECAR upon ME2kd. These findings led us to hypothesize that ME2 may play a crucial role in regulating the energy balance between the mitochondria and cytosol, and that compensation is reliant upon alternative pathways utilized by the cell. Such a role could explain the reciprocal changes observed in OCR and ECAR and the highly variable response to ME2kd.

Multiple experiments in this study, including glucose-labeled GC-MS, Seahorse flux analyses, western blotting, and gene expression, indicated a potential link between ME2 and *de novo* serine synthesis. Three specific observations from the GC–MS data support this idea: (i) malate concentrations differed substantially under ME2kd, yet labeling data did not indicate equally large flux changes; (ii) glutamate levels shifted in opposite directions to aspartate; and (iii) lactate synthesis was significantly reduced in ME2kd cells.

Initially, we thought that increased mitochondrial biogenesis was responsible for increased malate levels. However, our data from Seahorse suggests otherwise. We considered NAD^+^/NADH balance, which is a common thread across all pathways of interest. ME2 generates NAD(P)H from NAD(P)^+^, lactate dehydrogenase (LDH) recycles NADH to NAD^+^, and phosphoglycerate dehydrogenase (PHGDH) consumes NAD^+^ to produce NADH during serine synthesis. Finally, the malate–aspartate shuttle, which involves malate, aspartate, glutamate, and α-ketoglutarate, facilitates the transfer of reducing equivalents between the mitochondria and cytosol.

The observed potential connections between ME2kd, glycolysis, and *de novo* serine synthesis prompted us to examine its growth under nutrient-restricted conditions. We hypothesized that TNBC cells deficient in ME2 would exhibit heightened sensitivity to nutrient depletion compared with controls. Indeed, our results revealed distinct vulnerabilities in TNBC cells upon nutrient depletion (Fig. 6). MDA-MB-468 and Hs578T cells exhibited growth impairment in the absence of serine and glycine (Fig.6A), which lined up with labeling changes during serine deprivation (Fig. 5). However, BT-20 experienced inhibited growth in these conditions, with no changes to labeling, suggesting that serine uptake is integral for growth in this cell line. Glucose deprivation tests (Fig. 6B) were consistent with respect to Seahorse GST (Fig. 3) for MDA-MB-468and BT-20, while HCC70 showed an inverted correlation. The correlation between glucose deprivation sensitivity and altered glycolytic flux, as indicated by Seahorse data, highlights glycolysis as a potential vulnerability under ME2kd in some TNBC cell lines.

An important observation from these experiments is the differential reliance of TNBC cell lines on serine/glycine versus glucose. Hs578T and HCC1806 demonstrated greater dependency on serine and glycine availability, experiencing pronounced growth reductions under these conditions (Fig.6A), aligning with reduced serine labeling in both their controls and ME2kds under serine/glycine deprivation (Fig. 5), and a significant decrease in serine labeling following ME2kd in complete media (Fig. S3). HCC70 has been previously characterized with robust serine biosynthesis flux, while MDA-MB-468is known for increased serine pathway gene expression. This metabolic preference correlated with higher baseline lactate production and minimal labeling shifts in lactate and pyruvate upon ME2kd (Fig. 5 and Fig. S3), consistent with an inherently robust glycolytic pathway [13].

We hypothesized that the MAS could interconnect ME2, glycolysis, and serine biosynthesis. Specifically, we postulated that ME2kd leads to the accumulation of mitochondrial NAD^+^, thereby enhancing MAS activity and increasing cytosolic NAD^+^ levels for PHGDH-driven serine synthesis. Elevated cytosolic NAD^+^ and reduced malate-to‐pyruvate conversion would shift the LDH reaction in the reverse direction, preserving pyruvate for the TCA cycle. This conceptual model unifies the observed shifts in malate, aspartate, glutamate, and lactate by highlighting the central role of NAD^+^/NADH homeostasis and the malate–aspartate shuttle in coordinating metabolic pathways under ME2kd. This idea is further supported by a recent study by Broeks et al. (2023), who highlighted the significance of the malate-aspartate shuttle in *de novo* serine synthesis.

MDA-MB-468 and HCC70 showed profound growth inhibition under MAS inhibition, suggesting that MAS is highly active and plays a critical role in maintaining NAD^+^/NADH balance in these cells (Fig. 6D), concordant with the elevated serine labeling observed in these cells. In contrast, HCC1806 was largely unaffected by MAS inhibition alone, but showed pronounced sensitivity to the combination with ME2kd, indicating that ME2 plays a proportionally greater role in redox maintenance in these cells. Notably, neither MAS inhibition nor ME2kd had a significant impact on the normal-like cell line MCF10A growth, implying a reduced reliance on these pathways by non-tumorigenic cells. Collectively, these results suggest an interdependence between ME2 and MAS in regulating metabolic flexibility and highlight their potential as therapeutic targets for TNBC.

X-ray crystallography structural data combined with in vitro mitochondrial activity assays demonstrated direct target engagement between NPD-389 and ME2 (Fig. 7A-D). The NPD-389/ME2 complex structural data showed binding within the active site with coordinate bonds that chelate the magnesium metal center with extensions into a small non-polar pocket and toward the solvent space (Fig. 7B). Interestingly, the active site magnesium cation appears only in conjugation with either malate or NPD-389 binding, but is otherwise absent in complexed ME2/NAD + structures, even in the presence of high MgCl_2_ (10 mM) concentrations. Therefore, it is possible that NPD-389 coordinates the metal outside of ME2 and integrates it within the active site in the same manner that ATP and Mg^2+^ are considered constitutive complexes within the cell [42]. This binding mode of NPD-389 is consistent with its action as a competitive inhibitor with respect to the substrate malate [37]. Pharmacological inhibition of the malic enzymes using NPD-389 evoked cell viability decreases in some TNBC cell lines, but notably not in MDA-MB-468 (Fig. 7E). These effects were variable and cannot be attributed to the inhibition of ME2 directly, due to NPD-389’s lack of specificity for ME2 only.

NPD-389 effectively attenuated malate-ME2-driven respiration in mitochondria isolated from F-293 cells. This inhibition was dose-dependent and was restored to vehicle control levels upon the addition of pyruvate. Consistent with the reported IC_50_ for inhibition of recombinant human ME2 activity [37], 5 µM NPD-389 inhibited malate respiration ~ 50% relative to vehicle control. Increasing the NPD-389 concentration to 5-fold the IC_50_ (25 µM) inhibited 80% of malate respiration, which was again completely restored with the addition of pyruvate. Substrate-uncoupler-inhibitor-titration (SUIT) analysis further confirmed that inhibition of cellular respiration occurred through specific target engagement with ME2, as 25 µM NPD-389 did not interfere with the components of the electron transport chain. These findings show that ME2 mediates maximal complex 1 respiration with malate alone, and that its activity is ablated by NPD-389, consistent with the reported IC50, without affecting the components of the electron transport chain.

NPD-389, with its central quinone ring, is seemingly undesirable as a drug owing to its potential for futile redox cycling. However, the binding mode in ME2 suggested that this was not due to a problem within the active site. Given the high degree of conservation, the active sites of ME1/ME2/ME3 suggest that drug discovery efforts targeting these locations may not provide isoform selectivity. Whether targeting the active or dimer interface sites, computational modelling, including virtual screening, scaffold hopping, SAR assays, and chemical synthesis, will provide a path towards discovering novel malic enzyme inhibitors. Given the observed metabolic compensation via MAS and serine biosynthesis, future studies should explore combination therapies that target both ME2 and these compensatory pathways. Evaluating the efficacy of ME2 inhibition alongside MAS or PHGDH inhibitors could reveal whether dual-metabolic targeting enhances TNBC vulnerability.

## Conclusions

Our results highlight the role of malic enzyme 2 (ME2) in modulating TNBC progression through multiple metabolic and phenotypic effects. ME2 knockdown impaired the proliferation and/or migration of TNBC cell lines in a variable manner. ME2 knockdown significantly reduced tumor growth and extended survival in MDA-MB-468. Mechanistically, our transcriptomic and metabolic flux analyses revealed that ME2 knockdown disrupts energy homeostasis by altering key pathways, such as MAS and glycolysis, as well as *de novo* serine biosynthesis. Reduced ME2 activity appears to affect NAD+/NADH balance, which in turn affects the metabolic branch points in pyruvate-to-lactate conversion, α-ketoglutarate/glutamate interconversion, and serine synthesis. Notably, some cell lines rely on MAS to compensate for ME2 knockdown, suggesting that combination strategies targeting both ME2 and MAS could overcome adaptive metabolic flexibility for those tumors. Dependence on serine and glycine varied among cell lines, implicating that TNBC cells can pivot between amino acid uptake and *de novo* serine biosynthesis. These findings suggest that ME2 supports TNBC cell survival and metastatic potential by modulating metabolic and signaling pathways.

We provide evidence for a link between ME2 activity and the serine biosynthesis pathway, a pathway integral to the synthesis of nucleotides and amino acids that are vital for cell proliferation. Serine biosynthesis intersects with glycolysis, and its products feed into the folate cycle and methionine metabolism, thereby impacting cellular methylation processes and redox balance [43]. Low-serine diets have been analyzed as a treatment strategy in combination with chemotherapy or alone for TNBC [44]. Inhibition of ME2 could synergize with therapies aimed at other metabolic pathways, including MAS or de novo serine biosynthesis.

Pharmacological studies using NPD-389 further support the importance of the malic enzymes in TNBC growth. This pan-malic enzyme inhibitor blocked malate-driven respiration in a dose-dependent manner by engaging the active site of ME2 and coordinating its essential magnesium ion. Structural analyses confirmed that while NPD-389 can inhibit ME2 effectively, achieving isoform selectivity among ME1/ME2/ME3 may prove challenging. Nonetheless, novel strategies aimed at either the active site or dimer interface, along with rational drug design and structure-guided optimization, provide exciting avenues for future inhibitor development.

Overall, this work demonstrates that ME2 is involved in the metabolism of triple-negative breast cancer (TNBC), supporting tumor growth and survival through its involvement in energy production. Targeting ME2 in TNBC patients could disrupt metabolic homeostasis and improve therapeutic outcomes, potentially in combination with interventions targeting serine metabolism, glycolysis, or MAS. Further in-depth work on drugging compensatory mechanisms and metabolic plasticity among different TNBC subtypes will be critical for refining targeted therapies and improving patient outcomes, as well as for identifying new small-molecule inhibitors targeting ME.

## Supplementary Information

Supplementary material 1.

## Data Availability

No datasets were generated or analysed during the current study.
