# Supplementary material for: Interplay between malic enzyme 2, de novo serine synthesis, and the malate-aspartate shuttle drives metabolic adaptation in triple-negative breast cancer
Source: Cancer Metab. 2025 Oct 14;13:42. doi: 10.1186/s40170-025-00410-5 (PMC12523003; doi:10.1186/s40170-025-00410-5)
Supplement: Supplementary file 1 — Supplementary material 1. [file 40170_2025_410_MOESM1_ESM.pdf]

Supplemental Information

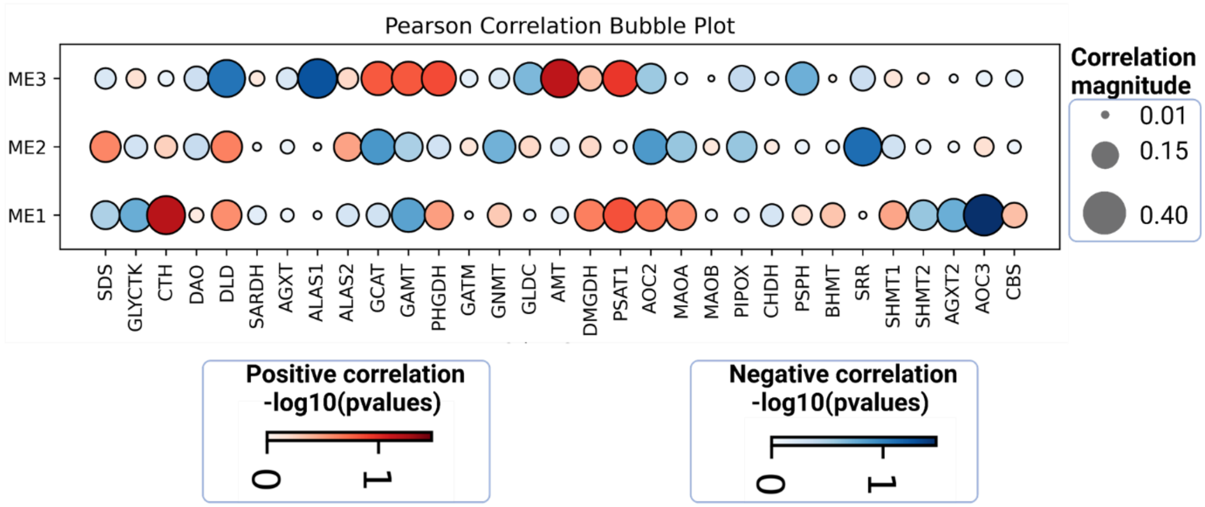

Figure S1: Bubble plot reflecting the Pearson correlation between the expression of malic enzyme paralogs (ME1, ME2 and ME3) and most genes in the serine and glycine metabolic pathways. The size of the bubbles indicates the strength of the correlation. Red bubbles are positive correlations while blue bubbles are negative correlations. The color scale for both the positive and negative correlations indicate the –log10(p-value) of the correlation. The data were obtained from Breast Invasive Carcinoma (TCGA, Cell 2015) that was deposited in cBioPortal (www.cbioportal.org). Only samples corresponding to Triple Negative Breast Cancer (TNBC) were used in the analysis (see methods for details).

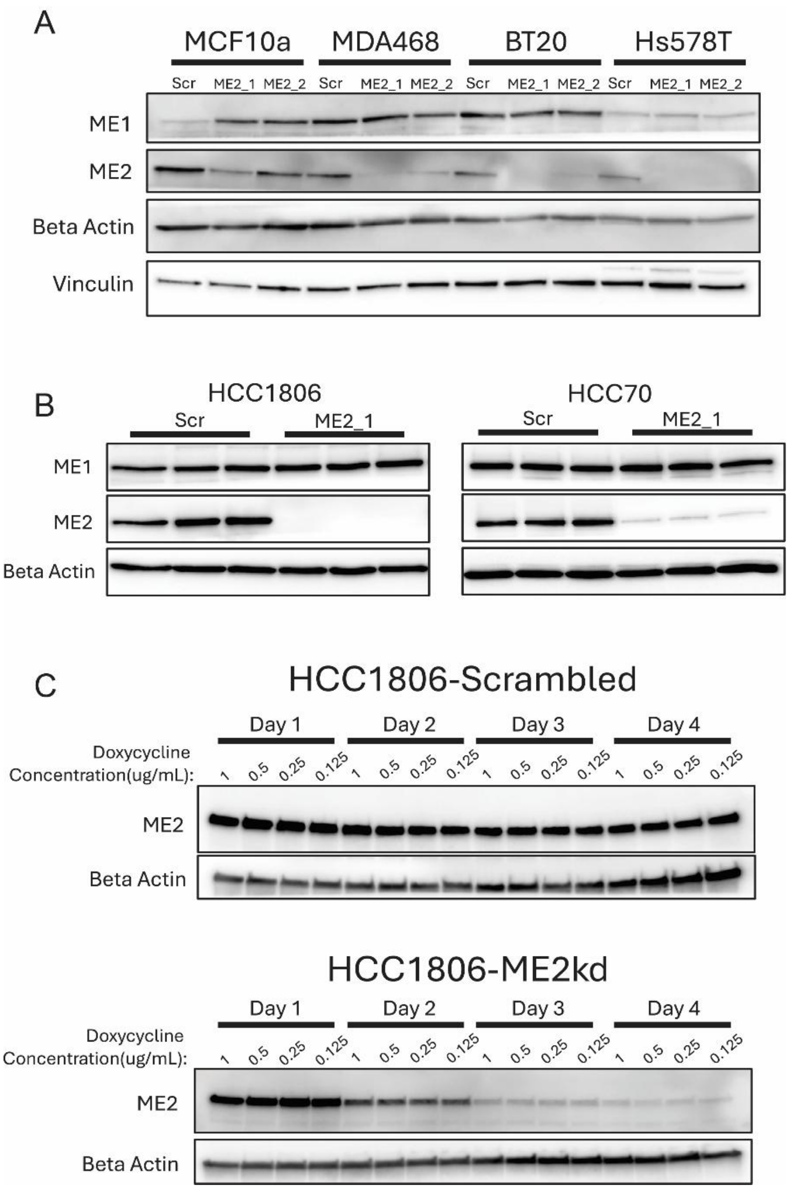

Figure S2: (A) Western blotting of proteins from MCF10a, MDA-MB-468, BT20 and Hs578T expressing scrambled (Scr) or two different ME2-targeting shRNAs (ME2_1; ME2_2), confirming knockdown of ME2 while examining ME1 expression. Beta Actin and Vinculin serve as loading controls. (B) Western blotting of proteins from HCC1806 and HCC70 expressing scrambled (Scr) or ME2-targeting shRNA (ME2_1). (C) Doxycycline dose response showing activation of the inducible promoter with various concentrations (1, 0.5, 0.25 and 0.125 ug/mL).

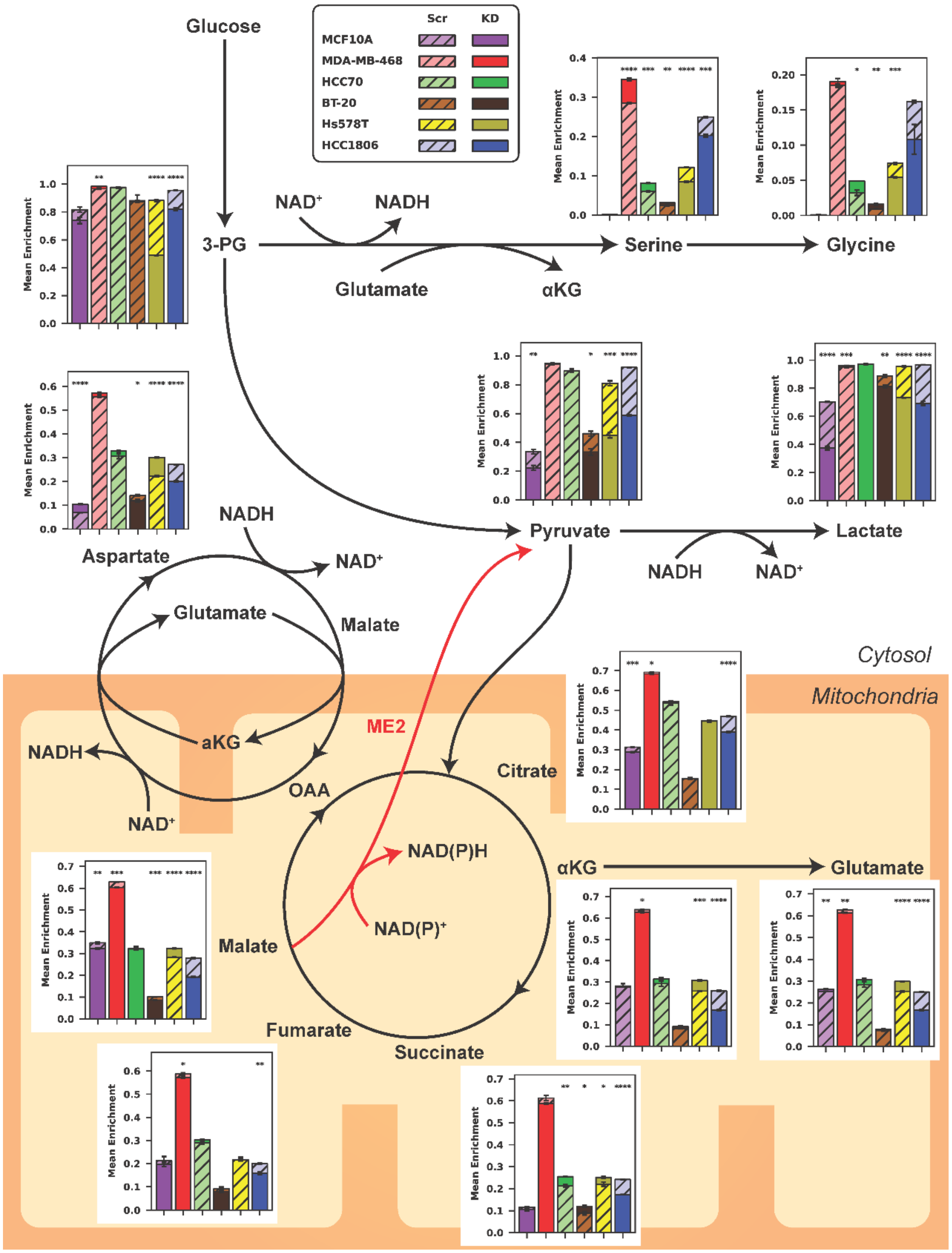

Figure S3: GC-MS mean enrichment data for central carbon metabolism under complete media condition. Each bar represents mean enrichment ratio of each metabolite in either the scrambled cell line (n = 3) or the ME2kd cell line (n = 3) overlapped on top of each other. All graphs depict standard error. All raw peaks were normalized using norvaline as an internal standard. Bottom-most circle in the mitochondria represents the TCA cycle and the circle in the center-left across the mitochondria and cytosol represents the MAS.

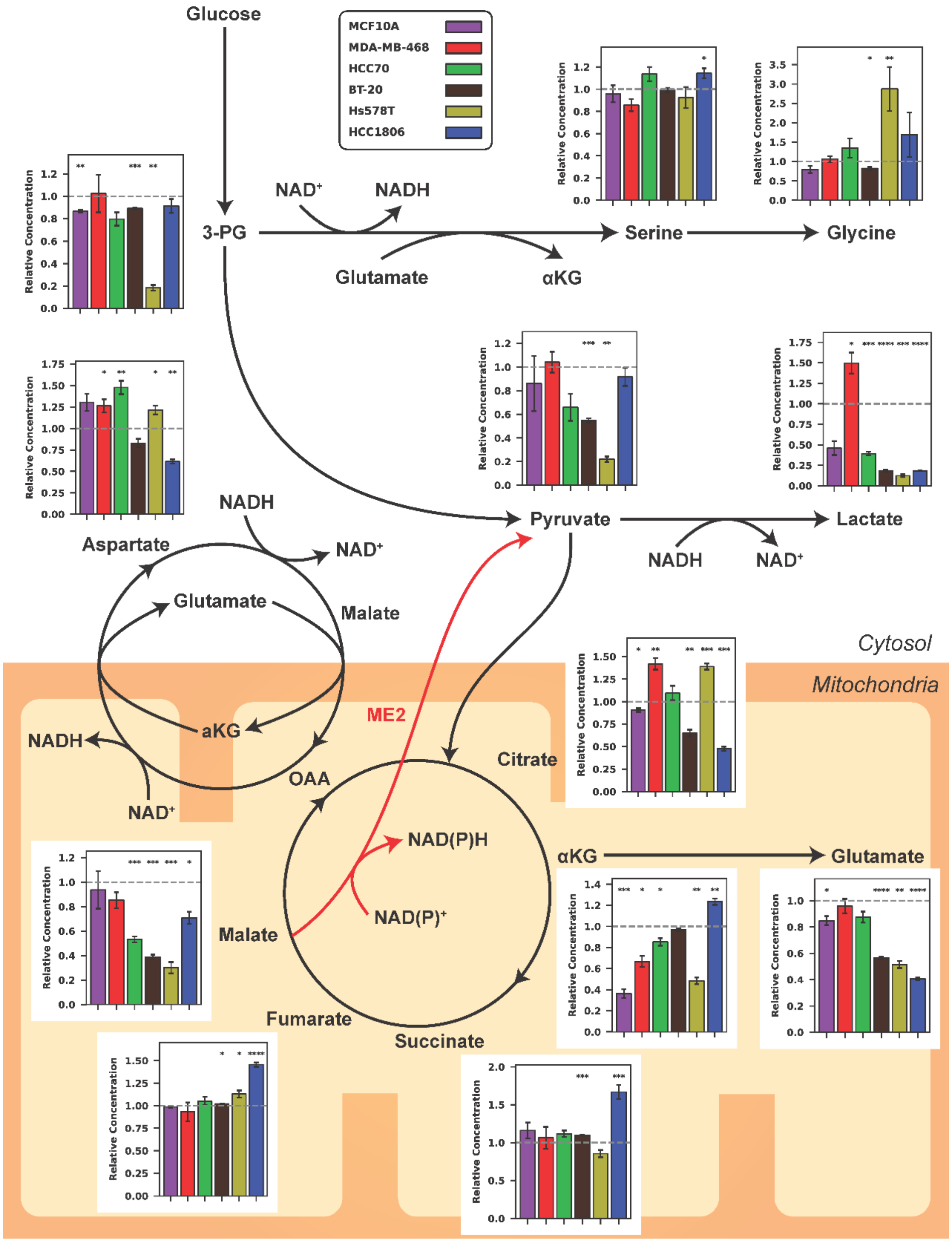

Figure S4: GC-MS concentration data for central carbon metabolism under serine and glycine deprived media condition. All bars represent concentration of each metabolite in the ME2kd cell line (n = 3) normalized by that in the scrambled cell line (n = 3) for each cell line. All graphs depict normalized and error-propagated standard error bars. All raw peaks were normalized using norvaline as an internal standard. Bottom-most circle in the mitochondria represents the TCA cycle and the circle in the center-left across the mitochondria and cytosol represents the MAS.

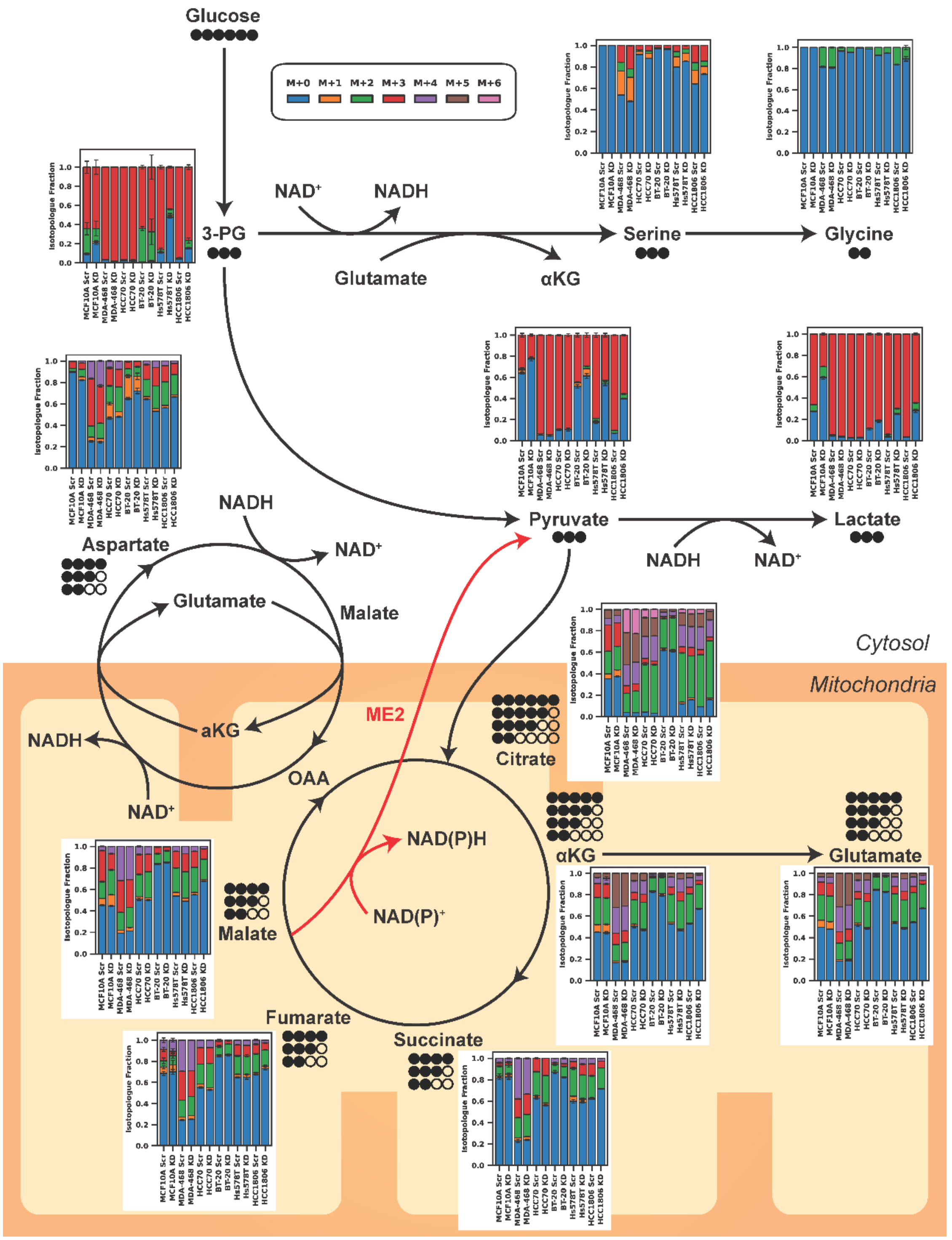

Figure S5: GC-MS mass isotopologue distribution (MID) of central carbon metabolism under complete media conditions. Bars represent corrected mass fractions for scrambled or ME2kd cell lines (each n=3). Data normalized to norvaline internal standard; error bars represent SEM. Circles indicate TCA cycle (bottom mitochondria) and MAS pathway (left, mitochondria-cytosol interface).

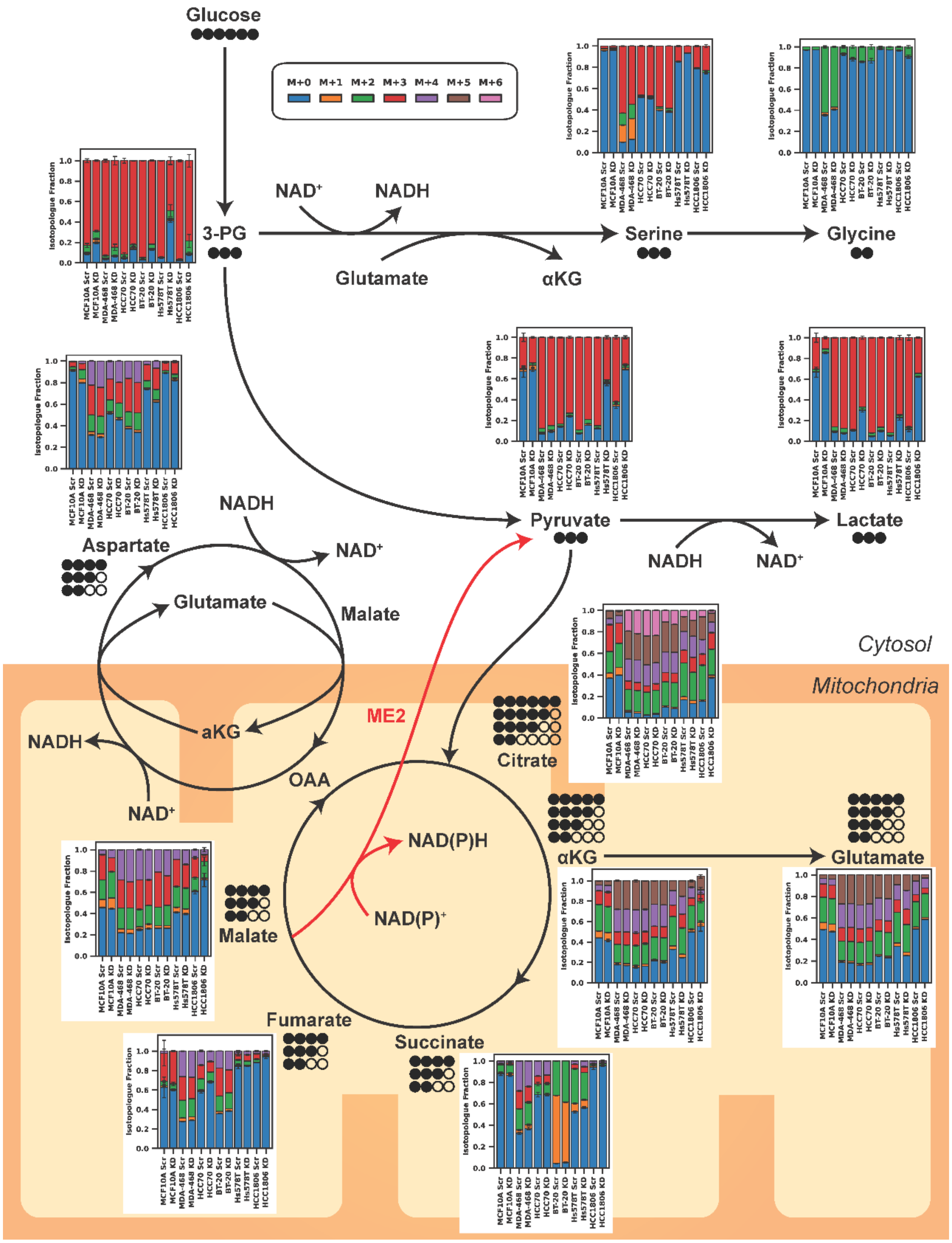

Figure S6: GC-MS mass isotopologue distribution (MID) of central carbon metabolism under serine/glycine-deprived conditions. Bars represent corrected mass fractions for scrambled or ME2kd cell lines (each n=3). Data normalized to norvaline internal standard; error bars represent SEM. Circles indicate TCA cycle (bottom mitochondria) and MAS pathway (left, mitochondria-cytosol interface).

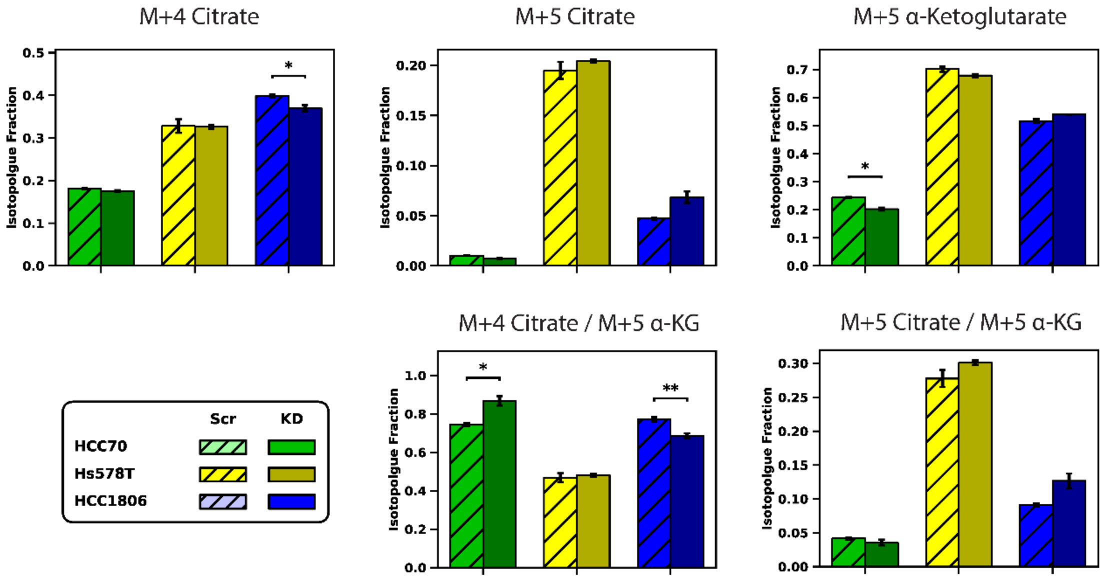

Figure S7: Mass isotopologue fraction of 13C5-glutamine-labelled M+4 Citrate, M+5 Citrate, M+5 α-KG, M+4 Citrate/M+5 α-KG, M+5 Citrate/M+5 α-KG.

Figure S8. Inhibition of malate respiration (mal-ADP) by 5 µM (A) and 25 µM (B) NPD-389 in F-293 mitochondria. Grey and black lines indicate inhibition by NPD-389 relative to vehicle control. Additional titrations show NPD-389 inhibition was specific to malate respiration, as the addition of pyruvate (pyr) restored Complex I respiration to vehicle control levels. Glutamate (glut) was added for maximal Complex I respiration. Complex 2 (succinate, succ) and uncoupled respiration (FCCP) were also unaffected by NPD389. Poisons have similar effects in control and NPD-inhibited mitochondria, including the Complex I inhibitor, rotenone, the ATP-synthase inhibitor, oligomycin (olig), and the Complex IV inhibitor, antimycin-a.

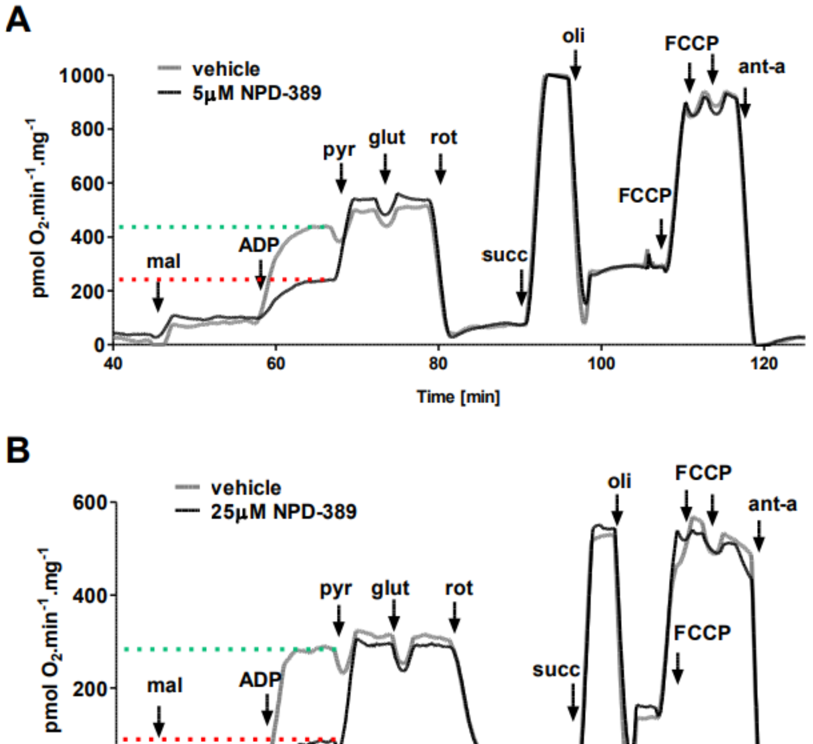

Validation of malate respiration inhibition with NPD-389 in F-293 mitochondria.

A comprehensive substrate-uncoupler-inhibitor-titration (SUIT) protocol was employed to validate the inhibition of malate respiration by NPD-389 and investigate whether it interfered with other components of the ETS. Malate respiration in mitochondria isolated from F-293 cells was inhibited by NPD-389 in a dose-dependent manner and restored to vehicle control levels upon addition of pyruvate (Fig. S8A, B). The IC50 concentration of NPD-389 for ME2 activity (5 μM) reported by Wen et al. (2014) inhibited malate respiration ~50% relative to vehicle control (Fig. S8A). Increasing NPD-389 concentration to 5-fold the IC50 concentration (25 μM) inhibited 80% of malate respiration, which was again completely restored with the addition of pyruvate (Fig. S8B). This finding implied that NPD-389 at concentrations <25 μM specifically inhibited malate-mediated respiration through ME2, but other components of the ETS were unaffected by the saturating concentrations used in the SUIT protocol.

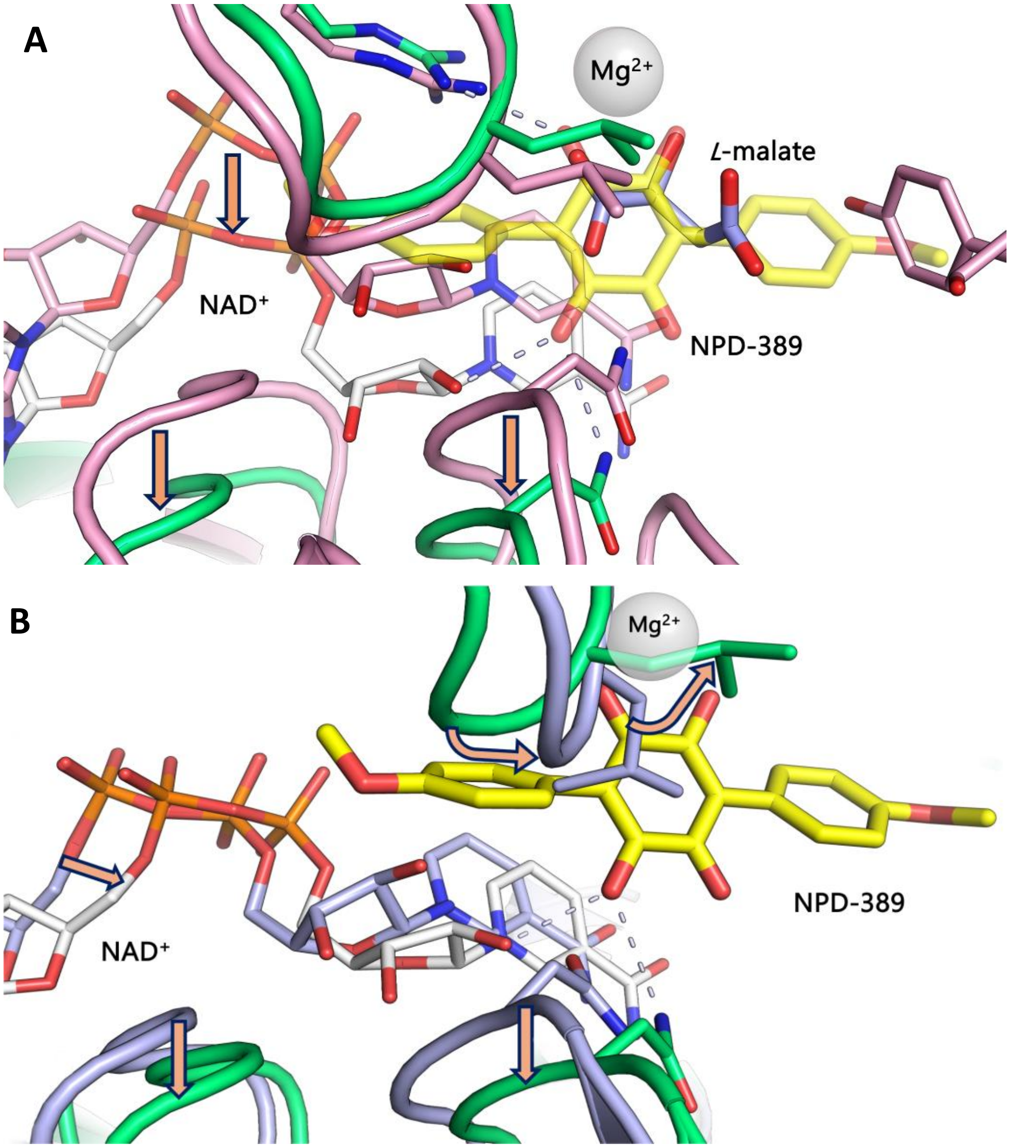

Figure S9: Crystal structure overlays showing NPD-389 induced movement in the malic enzyme 2 active site relative to the *L*-malate bound (A) and apo (B) structures. Significant movement of cofactor, side chains and protein, which could not be predicted from existing crystal structures, is indicated by red arrows. NPD is shown as a yellow stick model and is paired with an NAD^+ molecule in white and a protein model in green. The comparator malate structure is colored pink (protein and NAD+) and magenta (malate), and the apo structure is light blue (protein and NAD+). Selected side chains and loops are labeled, and selected hydrogen bonds are shown as thick dashed lines. Drawn using PDB structures 1PJ2 and 9AYI using PyMOL (Schrödinger, Inc.).

	ME2/NAD/NPD-389	ME2/NAD
Diffraction source	MX2, Australian Synchrotron	MX2, Australian Synchrotron
Wavelength (Å)	0.95370	0.95370
Temperature (K)	100	100
Detector	DECTRIS EIGER X16M	DECTRIS EIGER X16M
Total rotation range (°)	360.0	720.0
Exposure time (s)	36	72
Space group	*C*2	*P*1
a, *b*, *c* (Å)	204.05, 58.92, 106.93	74.33, 98.17, 107.74
α, β, γ (°)	90.00, 101.94, 90.00	65.32, 70.75, 74.89
Mosaicity (°)	0.12	0.12
Resolution range (Å)*	49.17 – 2.45 (2.54 – 2.45)	47.37 – 1.89 (1.93 –1.89)
Total No. of reflections*	554735 (54877)	729393 (28498)
No. of unique reflections*	46166 (4486)	200119 (8176)
Completeness (%)*	100 (100)	97.2 (79.9)
Multiplicity*	12 (12.2)	3.6 (3.5)
$\langle I/\sigma(I)\rangle$*	9.0 (0.9)	6.9 (1.6)
$R_{\text{p.i.m.}}$*	0.084 (1.814)	0.068 (0.745)
$CC_{1/2}$*	0.998 (0.595)	0.990 (0.483)
Wilson *B* factor ($Å^2$)	51.0	28.1

*values in parentheses are for the outer shell.

Table S1: X-ray data collection and processing

Table S2. X-ray crystal structure solution and refinement

Values in parenthesis are for the outer shell

	**ME2/NAD/NPD-389**<br>**PDB ID 9MYF**	**ME2/NAD**<br>**PDB ID 9AYI**
Resolution range (Å)	49.22 – 2.45(1.18 – 1.15)	47.42 – 1.89 (1.54 – 1.50)
Completeness (%)	100	97.1
No. of reflections, working set	43784 (3756)	189840 (12285)
No. of reflections, test set	2378 (195)	10256 (713)
Final R_{cryst}	0.181 (0.440)	0.177 (0.286)
Final R_{free}	0.278 (0.442)	0.201 (0.279)
**No. of non-H atoms**		
Protein	8747	17347
Water	74	1961
Ions	7	6
Ligands	168	412
Total	9018	20074
**Average *B* factors (Å^2)**		
Protein	69.2	21.0
Water	53.3	42.6
Ligands	70.5	49.1
**Root mean square deviation**		
Bond lengths (Å)	0.002	0.003
Angles (°)	0.962	1.094

	ME2/NAD/NPD-389 PDB ID 9MYF	ME2/NAD PDB ID 9AYI
**Validation**		
Ramachandran most favored (%)	96.4	97.7
Sidechain rotamer outliers (%)	0.1	0.3
Clash score	2	2

Malic enzyme 2 cloned sequence.

MLHIKEKGKPLMLNPRTNKGMAFTLQERQMLGLQGLLPPKIETQDIQALRFHRNLKKMTSPLEKYIYIMGIQERNEKLFY
RILQDDIESLMPIVYTPTVGLACSQYGHIFRRPKGLFISISDRGHVRSIVDNWPENHVKAVVVTDGERILGLGDLGVYGMG
IPVGKLCLYTACAGIRPDRCLPVCIDVGTDNIALLKDPFYMGLYQKRDRTQQYDDLIDEFMKAITDRYGRNTLIQFEDFGN
HNAFRFLRKYREKYCTFNDDIQGTAAVALAGLLAAQKVISKPISEHKILFLGAGEAALGIANLIVMSMVENGLSEQEAQKK
IWMFDKYGLLVKGRKAKIDSYQEPFTHSAPESIPDTFEDAVNILKPSTIIGVAGAGRLFTPDVIRAMASINERPVIFALSNPT
AQAECTAEEAYTLTEGRCLFASGSPFGPVKLTDGRVFTPGQGNNVYIFPGVALAVILCNTRHISDSVFLEAAKALTSQLTDE
ELAQGRLYPPLANIQEVSINIAIKVTEYLYANKMAFRYPEPEDKAKYVKERTWRSEYDSLLPDVYEWPESASSPPVITELEH
HHHHH

Methods (Supplemental)

Mitochondrial isolation from F-293 cells.

For high-resolution respiratory studies, mitochondria from F-293 cells were isolated using differential centrifugation. Cells were spun at 650 × g for 5 min at 4°C, and the pellet was resuspended in 1.5 mL of 10 mM Tris-HCl, 250 mM sucrose, 0.5 mM EDTA, pH 7.4 (MIB). Cells were manually homogenized by 30-40 strokes in 2 mL Potter-Elvenjhem and the homogenate was centrifuged at 1000 × g (10 min at 4°C). The supernatant was transferred to a 1.5 mL tube, and the pellet was resuspended in 750 µL MIB and centrifuged again at 650 × g (10 min, 4°C). Supernatants were spun at 14,000 × g (10 min, 4°C) and pellets were resuspended in 30 µL of 60 mM K-lactobionate, 20 mM HEPES, 110 mM sucrose, 10 mM KH2PO4, 20 mM taurine, 0.5 mM EGTA, 3 mM MgCl2, and 1 mg/ml bovine serum albumin, pH 7.1 (MiR05).

Inhibition of malate respiration by the ME2 inhibitor NPD389 using high-resolution respirometry.

High-resolution respirometry was performed using OROBOROS Oxygraph-2K (Innsbruck, Austria) at 37°C in 2 mL of MiR05. The air saturation of the medium was 215 nmol O2.mL-1 at a barometric pressure of 95 kPa. Mitochondria from F-293 cells (0.1 mg) were added to each of the six oxygraph chambers, followed by either 5 or 25 µM NPD-389, where the respective adjacent chamber acted as a vehicle control (DMSO). Following 15 min of incubation to allow depletion of intra-mitochondrial substrates, malate (2 mM) and ADP (1.25 mM) were added, followed by pyruvate (10 mM) and glutamate (10 mM) for maximal C1 respiration. Rotenone (1 µM) was added to inhibit complex one respiration, then complex two respiration was then stimulated with succinate (10 mM). ATP synthase was inhibited with oligomycin (5 µM) and maximal electron transport flux was measured by uncoupling mitochondria with 0.5 µM carbonyl cyanide 4-(trifluoromethoxy)phenylhydrazone (FCCP). Background respiration was measured using antimycin-a (2 µM), which was subtracted from other respiration measurements.
